# Design of a Learning Development Program to Support First-Year Undergraduate Medical Students in the Transition to a PBL Curriculum

**DOI:** 10.1007/s40670-023-01790-3

**Published:** 2023-05-04

**Authors:** Masego B. Kebaetse, Brigid Conteh, Maikutlo Kebaetse, Gaonyadiwe George Mokone, Oathokwa Nkomazana, Mpho S. Mogodi, John Wright, Rosemary Falama, Kalman Winston

**Affiliations:** 1 Department of Medical Education, University of Botswana, Gaborone, Botswana; 2 Communication and Study Skill Unit, University of Botswana, Gaborone, Botswana; 3 Department of Biomedical Sciences, University of Botswana, Gaborone, Botswana; 4 Department of Surgery, University of Botswana, Gaborone, Botswana; 5 Department of Public Health and Primary Care, Cambridge University, Cambridge, UK

**Keywords:** First-year transition, Learning development, Curriculum design, Academic support

## Abstract

While the evaluation of learning development interventions needs to be considered carefully and included at the curriculum design stage, there is limited literature on the actual design of interventions, especially on how these designs evolve and improve over time. This paper describes the evolution of a learning development program intended to support first-year medical students adjusting to a problem-based learning curriculum. We used a design-based research approach, articulating our theoretical grounding and incorporating students’ voices to develop an “optimal” intervention for the specific challenges in our context. We describe lessons learned around four aspects: students’ growth and development, teachers’ professional growth and development, program design principles, and the emergent components of a learning development program. Overall, our students describe the Learning Success Program as adding value by enabling the adoption of a repertoire of skills and strategies for learning management. Additionally, the incremental nature of design-based research allowed for the development of a context-specific program that considers students’ voices through needs assessment and feedback on the program offerings. It has also provided an opportunity for the professional development of teachers through feedback from classroom practice, reflection, and the literature.

**Supplementary Information:**

The online version contains supplementary material available at 10.1007/s40670-023-01790-3.

## Introduction

The highly competitive admissions process through which most students enter medical school assumes they come capable and prepared. Despite the history of prior high academic performance and learning success, some medical students may later experience academic and personal difficulties that lead to learning challenges and failure [1, 2]. Growing medical education learning development (learning-to-learn or learning support) literature points to ongoing efforts to address medical students’ learning challenges [3]. Traditionally, learning development interventions have focused on students who failed (deficit-reactive approach) [2] or on students perceived as having an inherent deficit before failure (deficit-proactive approach) [4]. Deficit approaches are essential in getting students back on track throughout medical training. However, such approaches may overlook other students who could benefit from learning development, such as those who are marginally passing [2]. In contrast, a positive-proactive developmental approach focuses on stimulating all students’ holistic development alongside their academic and clinical competence [2].

Since students are likely to experience challenges when they lack the necessary knowledge, skills, and attitudes, proactive developmental support focuses on enabling reasonable adjustment to the learning environment by providing a “buffer” to scaffold students’ learning [2, 5] This approach goes beyond remediating failure to facilitating students’ personal and professional formation [2]. Proactive developmental support is especially critical in the first year of problem-based learning (PBL) undergraduate medical training because such a program requires a higher degree of self-regulation than prior schooling where teaching and learning were largely teacher-centered. As such, intervention in the first year of medical school equips students to make the necessary learning changes and disrupts the pattern of failure experienced by many struggling students [6].

A recent review of learning development interventions for first-year medical students has highlighted three main intervention components: academic or learning skills such as reading, content aspects such as basic sciences, and personal and professional skills such as time management [4]. Some interventions also include medical program expectations and the culture of medicine [4]. Intervention duration varies from a few weeks to 2 years, with longer durations considered more beneficial [4] although the optimal blend of content and duration of the intervention is unclear [3].

Reviews of medical education learning development programs have identified several gaps worth considering in designing future interventions: the underlying theoretical or pedagogical grounding has not always been explicit [4, 6]; there has been a lack of complexity (multiple interrelated and interdependent components) [6]; and students’ voices are often missing [4]. Finally, there is limited literature on how intervention designs evolve over time, which could hinder the development of future interventions.

This paper describes the evolution of a positive-proactive learning development program for supporting first-year undergraduate medical students’ adjustment to a PBL curriculum. We used a design-based research approach to implement a learning development program for first-year medical students. We identified program components, articulated our theoretical grounding, and incorporated students’ voices to create an intervention for our context-specific challenges. We offer recommendations for essential components of an effective learning development program and some general principles for designing proactive learning development interventions for first-year undergraduate medical students.

## Study Setting

The Bachelor of Medicine Bachelor of Surgery (MBBS) program at the University of Botswana Faculty of Medicine is a 5-year undergraduate, integrated, hybrid PBL program. The first two years focus on biomedical sciences, public health, and basic clinical and communication skills. Paper-based clinical PBL cases drive the learning for each week. PBL tutorials are complemented by other learning events, such as self-directed learning, plenaries, bio-practicals, clinical placements, clinical and communication skills sessions, resource sessions, and workshops [7]. The program uses the Maastricht seven-jump PBL process [8].

During the formative years of the MBBS program, first- and second-year medical students submitted weekly written reflections intended to synthesize and integrate concepts from the week’s learning experiences. Two of the research team members, who were PBL facilitators, were dissatisfied with students’ poor integration of concepts and the lack of depth in responses to questions requiring higher-order thinking. Students were also observed as exhibiting “unproductive” behaviors, such as shortcutting the brainstorming process, rushing through PBL tutorials, not asking questions, not challenging one another’s thinking, and displaying a competitive attitude that stifled the open discussion needed for learning in the PBL tutorials. The team concluded that there was a need for a learning development program to support students’ adjustment during the first year. We named our local learning development program the Learning Success Program (LSP).

## Research Team

Although we believed that students needed support in adjusting to medical school, we started our research project when most of us were new medical educators with limited experience in student support beyond our content areas and our new medical school lacked the in-house expertise to provide guidance. As such, we sought to understand our students’ experiences in adjusting to the first year of medical school. Furthermore, we also needed to understand the literature extensively so that our preparatory program would be informed by best practices and be responsive to our context.

The research team (four male and five female members) comprised biomedical scientists and educationists at master’s and PhD levels, medical and public health specialists, and a fifth-year medical student. An educational consultant experienced in qualitative research was part of the team during the needs assessment portion of the study and conducted all the interviews. BG and KW joined the team after the pilot of the LSP when the team published the literature review manuscript. BG brought the experience of teaching English as a second language for many years and teaching in the Communications and Study Skills course offered to all first-year University of Botswana students. KW became the mentor for the team based on his extensive learning development work. The three biomedical scientists actively taught in the first year and were also PBL facilitators for the duration of the study. Although five team members had experience with PBL as facilitators and/or previously as students, we lacked a shared understanding of our students’ experiences in transitioning to a PBL program during their first year of medical training.

## Theoretical Underpinnings of the Intervention

Self-directed learning [9] (SDL) is closely associated with the implementation of PBL both as an educational objective and a method of curricular delivery [10] to groom self-directed life-long learners [11]. SDL skills and competencies, such as collaboration, professionalism, scholarship, and effective communication, are considered essential for an expert and safe doctor [11, 12]. Although SDL is sometimes conflated with self-regulated learning (SRL) [13], SDL is a broader (macro-level) concept: the development of SDL competencies of personal autonomy, self-management, learner control, and independent pursuit of learning [14] are realized through the enactment of core SRL skills (task analysis, self-efficacy, self-control, self-observation, self-judgment, self-reaction) [15, 16]. Contemporary practice broadens the understanding of regulatory learning beyond SRL to include co-regulated learning (CoRL) and socially shared regulated learning (SSRL) [17–19]. In CoRL, the interactions between team members mediate the regulatory work, so it is distributed and shared with capable others in the community [17, 18]. In SSRL, group members interdependently and collectively utilize regulatory processes to achieve a shared outcome [17, 18]. CoRL and SSRL processes are essential to fully realizing the power of group learning in a PBL context.

To identify the skills and competencies that could become components of the LSP, we drew upon aspects of SDL [9] and SRL [14–16]. In conceptualizing the structure and delivery of the LSP, we utilized constructivist learner-centered approaches, SSRL and CoRL elements in which students learn through small-group collaboration (e.g., developing shared goals and planning, mutual support, social processes, attention to interactions), opportunities for practice both in and outside the class session, peer and facilitator feedback, and reflection [18–22].

## Project Approach

We used design-based research (DBR), sometimes called design research, to design and implement a context-aligned learning development program. Plomp [23 p13] describes DBR as “the systematic study of designing, developing and evaluating educational interventions (such as programs, teaching–learning strategies and materials, products and systems) as solutions for complex problems in educational practice, which also aims at advancing our knowledge about the characteristics of these interventions and the processes of designing and developing them.” In DBR, the design of educational materials or interventions is the critical aspect of the research [24] with the intent to solve problems or make improvements [25]. DBR studies are informed by prior research, and involve a collaboration between researchers and practitioners [23]. DBR embraces the iterative and systematic nature of educational and instructional design processes involving analysis of practical problems (analysis), development of solutions (design), iterative cycles of testing and refinement (evaluation and revision), and reflection to produce design principles [23, 26]. We used the Consolidated Criteria for Reporting Qualitative Research checklist [27] to guide the reporting of our results related to qualitative data and especially to improve our reflexivity throughout the process.

Consistent with the rapid prototyping of instructional interventions [28], the iterative nature of DBR allowed us to (i) implement the intervention as it was being developed, (ii) embrace the complex adaptive nature of the intervention [23], and (iii) pursue more than one outcome such as students’ use of effective learning strategies and overall management of their learning process [29]. We obtained feedback from multiple cohorts [30, 31], identified learning challenges and the component skills to address the challenges, and made incremental changes over time. As a medical school in an African context, the use of DBR also enabled us to include content specific to our context, e.g., including “botho” (humanness evidenced by a humble, respectful, courteous, well-mannered demeanor) [32, 33] as part of professionalism.

**Fig. 1:**
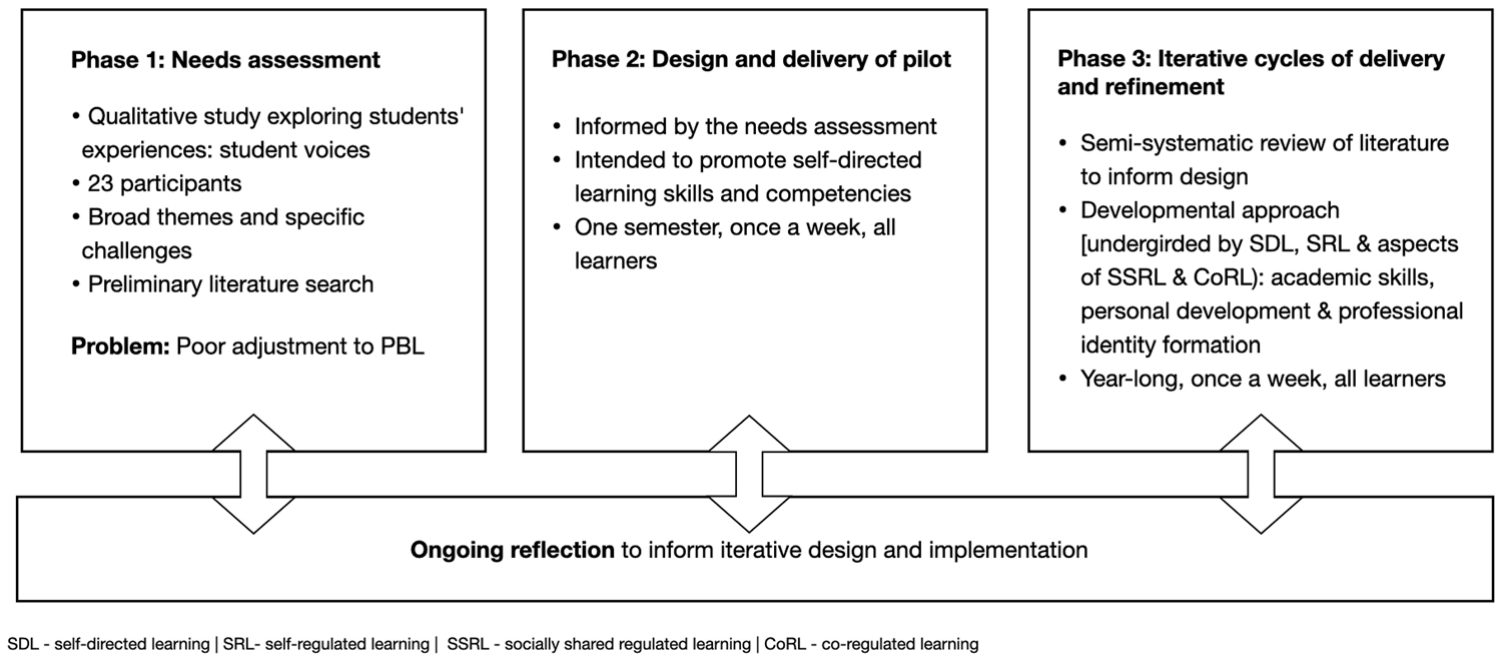
Iterative process of developing the Learning Success Program

We mapped our project using a four-component process [26] as shown in Fig. 1, considering the findings from four successive cohorts. During this time, neither the Bachelor of Science program from which students came nor the MBBS program itself had changed, and students had reported similar challenges across the four cohorts.

### Phase 1: Exploring First-Year Medical Students’ Learning Experiences During the First Semester of a PBL Program

A cohort of 51 first-year undergraduate medical students and their six PBL facilitators were invited to participate in a needs assessment study. Twenty-three students and five PBL facilitators (three of whom were study researchers) agreed to participate, signed consent forms, and completed the study. Participants ranged in age from 18 to 25 years. Fourteen came from the BSc program, seven from the pre-med program, and one student came from both A-levels and pre-med. Three international students had previously attended private senior secondary schools, while all the local students had attended public senior secondary schools.

Data were collected through non-participant observations and semi-structured in-depth interviews between October 2013 and March 2014. The findings of the non-participant observations were used to develop questions for the interviews. Interviews generally lasted 90 min, were audio-recorded, and transcribed verbatim by a research assistant. The consultant and another team member reviewed the transcripts against the audio recordings to ensure accuracy.

We used the six stages of the Braun and Clarke inductive reflexive thematic analysis framework [34, 35] to analyze our interviews — familiarization with data; generating initial codes and categories; searching for themes; reviewing, consolidating, and determining relationships between themes; defining, and naming themes; and writing the report. Thematic analysis enabled us to identify patterns in the data to understand our students’ experiences better. It allowed us to search for commonly shared behaviors, thoughts, and experiences [34, 36, 37].

Each team member read and re-read three transcripts for familiarization, generated initial codes through open coding, met to discuss the codes, and reached a consensus about what we found in the data. Subsequently, team members each read and coded at least two transcripts, while one team member read all the transcripts and consolidated the coding from the rest of the team. Next, we collated the various codes into potential themes and checked the codes to ensure that they were relevant to the theme and consistent with each other, checking themes for coherence. Then we defined and named the themes, making the statements as complete as possible [37]. Team members used regular debriefing to review the data and consider multiple perspectives.

The consultant was not part of the data analysis, interpretation, or manuscript writing processes. Although BG and KW joined the team after the data collection and analysis, they became involved in the ongoing interpretation and use of the data to revise and strengthen the LSP. Team members have different roles and participation in teaching first-year medical students and the LSP. For instance, while the rest of the team is from the medical school, the inclusion of BG and KW provided somewhat of an outsider’s perspective, which helped with objectivity and sense-checking [27]. Having different roles and viewpoints ensured that we are able to challenge each other thinking and bring different perspectives to the ongoing development of the LSP.

The following themes were identified: (a) students experience a range of learning challenges during the transition to the PBL process, (b) students’ attitude towards PBL is influenced by their understanding of the PBL process, (c) students who adopt self- and social regulatory study strategies feel more successful and less stressed, (d) some students see the benefit of working in groups while others prefer working alone, (e) students’ are uncertain about which resources to use and how best to appraise them, (f) many students failed to appreciate and or utilize the support available to them by the university, often turned to other sources for support, but acknowledge the need for a dedicated program with the medical school (Supplement A).

### Phase 2: Development and Delivery of the Learning Success Workshop Semester-Long Pilot Program

We analyzed the needs assessment report and reviewed the literature to determine the components, learning outcomes, and topics of a semester-long pilot program. The primary aim of the program was to enable students to develop and implement a strategy for effective learning in a learner-centered environment by addressing some of the learning challenges expressed in the findings. We focused mainly on learning from an individual, self-regulated learning perspective: the students’ understanding of their role from a learner-centered perspective [9] and the acquisition of various learning strategies to direct their learning [38], as shown in Table 1.

**Table 1:** Components, learning outcomes, and topics covered in the Pilot Learning Success Program

**Primary outcome**	**Component and learning outcomes**	**Topics**
Develop and implement a strategy for effective learning in a learner-centered environment	**Understanding yourself** Articulate the role of a student in a learner-centered environment	• Types of transition in medical school • What is learning — eight things needed to be an effective learner [39] • Teacher-centered vs. learner-centered practice, exploring prior and current learning contexts • Learning preferences and implications for learning • Learning with others
	**Learning strategies** Use effective study strategies for a learner-centered environment	• Learning strategies: minute paper, concept maps/mind maps • Self-management strategies: time management

The primary facilitators of the LSP (MBK and BG) developed three questions to be used for the feedback session at the end of the semester, which a colleague from the Department of Medical Education facilitated. All students (49) who happened to attend the last session of the LSP for the semester participated in the feedback session. The cohort had 60 students. Each student responded in writing to the following questions: (a) What has been your most significant learning this semester? (b) What is still unclear? (c) What would you still like to learn? Data were analyzed by MBK and BG using the same approach as in Phase 1. Some of the themes identified included (a) the attitude toward PBL is influenced by their understanding of the PBL process, (b) students who adopt self- and social-regulatory study strategies feel more successful and less stressed, and (c) students experience a range of learning challenges during the transition to the PBL process (Supplement A). A report on the 2016–17 LSP delivery (student feedback and facilitators’ reflections) was developed (Supplement A).

### Phase 3: Iterative Phases of Development, Delivery, and Refinement: 2017–2019

We analyzed the reports from the needs assessment and the pilot program to determine components, learning outcomes, and topics for the upcoming years. The focus of the program moved beyond the learners’ roles and responsibilities to include components aligned to SRL/SDL and SSRL/CoRL competencies and context-specific aspects during the next 2 years. For instance, we expanded the repertoire of skills for directing and regulating learning [38]. Also, the learning space was organized to support working in small groups as we emphasized collaborative learning [22] and the development of feedback literacy [40].

Consistent with the literature on dose–effect [3, 20] and feedback from the pilot, we expanded the program to span the whole academic year, which allowed us to add more topics and deeper focus. Sessions were held weekly with the entire first-year cohorts. To ensure relevance, learning activities such as concept mapping, pre-reading, and the minute paper utilized the biomedical content from the week’s theme for practice [3]. The first semester focused on learning strategies and collaborative learning, and the second semester focused on personal and professional skills. Sessions were primarily facilitated by two staff members (both educationists); other staff members (e.g., counselors and biomedical scientists) came as needed.

In addition to the components from the pilot program, the 2017–2018 program included *collaborative learning* and *self-management* (e.g., self-care and financial literacy). As the 2017–2018 LSP was being delivered, several ongoing projects impacted the future offering of the LSP. First, we conducted a semi-systematic review of the literature exploring learning development intervention programs for addressing learning challenges and deficits in the first year of medical school [4]. This informed further development of components and topics of the LSP. Second, the MBBS curriculum review report [41], finalized during 2017–2018, revealed several gaps in the curriculum delivery, one of which was related to *ethics and professionalism*, which the curriculum committee decided should be included in the LSP.

Finally, the Faculty of Medicine developed a student support and mentoring (SSM) program, and the LSP was formalized as part of that program [42]. The SSM program responded to anecdotally reported psycho-social (e.g., stress, alcohol, and drug abuse) and socio-economic (e.g., food insecurity) challenges faced by students. As a result, the self-management theme of LSP was expanded to include some aspects related to psycho-social matters.

At the end of each academic year, students (56 of 65 in 2017–2018 and 48 of 70 in 2018–2019) who happened to attend the last session of the LSP (attendance was voluntary throughout the LSP) were invited to participate in the feedback session. A colleague from the Department of Medical Education facilitated the session. Students responded to the following questions in writing: (a) What has been your most significant learning from the LSP this academic year? (b) What improvements, if any, have you made to enhance your learning during the year? MBK and BG developed the questions and analyzed the data using the same approach as in Phase 1. Some of the themes identified included the following: (a) students were uncertain about effective strategies to adopt for learning but were willing to be capacitated on learning how to learn effectively, (b) students recognize time management as essential for managing their learning and maintaining a healthy work-life balance, (c) students were uncertain about how to navigate the PBL process (Supplement A). A report of the LSP delivery for each academic year (student feedback and facilitators’ reflections) was developed (Supplement A).

### Ongoing Reflection

Critical reflection [43, 44] has been integral throughout the process, allowing us to examine our experiences and make necessary changes. The primary facilitators of the LSP have engaged in consistent conversational reflections using *The What? Model of structured reflection* [45]. The process has involved post-session, end-of-semester, and end-of-year reflections on our observations, review of students’ feedback, journal entries, and continued literature review. For instance, although students found learning style inventories helpful for self-introspection, their use has been challenged in the literature [46, 47], leading us to minimize their role and utilize them as tools for reflection.

### Summary of the DBR Process

**Fig. 2:**
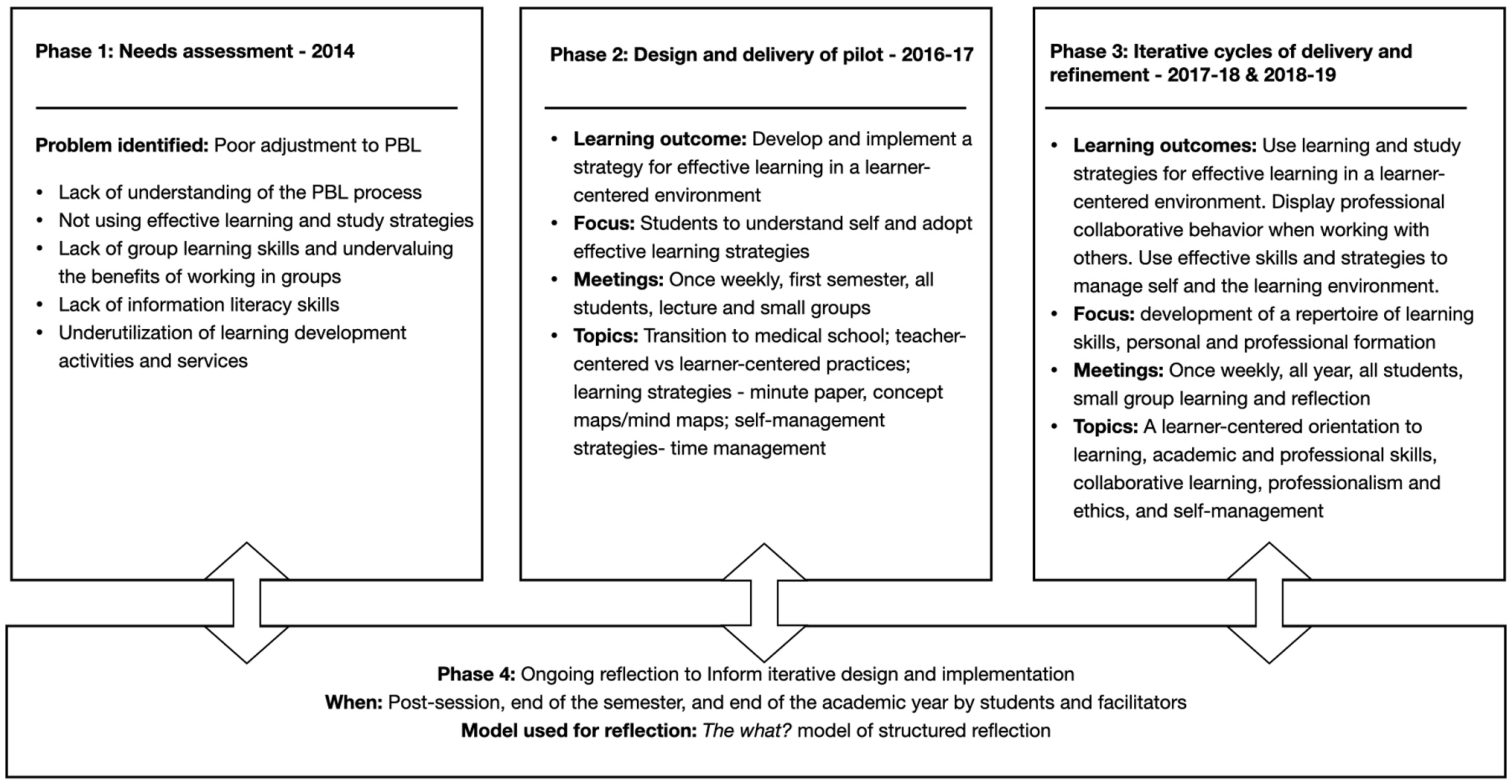
DBR process for develping a learning development program for first year undergraduate medical students

The iterations of our LSP are summarized in Fig. 2, highlighting the main changes that have occurred in the various phases of our design and implementation.

## Lessons Learned

This section describes lessons learned around four main aspects identified in our process: students’ growth and development, teacher professional growth and development, program design principles, and the emergent LSP.

### Students’ Perceptions of Growth and Development

Some students entering medical school continue to lack the self-regulatory skills needed for academic success [6, 48]. Our students experienced learning challenges commonly described in the literature, including poor time management and inadequate effort, poor integration of concepts, discomfort and reluctance to work in groups, lack of clarity about the depth to which they had to study, and unfamiliarity and challenges with the PBL process. These learning challenges have been associated with poor study and critical thinking skills [48], challenges with metacognition and self-regulation [48], and the inability to transition to self-directed learning [49]. Students also raised concerns about being stressed, not maintaining a healthy work-life balance, and whether the additional effort would improve their grades.

Students across all cohorts were clear about the intent of the LSP in supporting them to acquire skills and strategies for learning in medical school and beyond. Additionally, students reported an enhanced understanding of their role in a learner-centered PBL curriculum. They found it worthwhile to adopt self-management strategies such as time and stress management. Moreover, they acknowledged the benefits of utilizing a toolkit of learning strategies, such as concept mapping, reflection, minute papers, and muddiest points. Their increased comfort concerning working in small groups is an essential outcome considering our PBL curriculum. Most students were open to learning new skills and strategies, indicating that the LSP afforded them the academic support they needed for learning. Nonetheless, consistent with what has been reported elsewhere [21, 39], a few students were not altogether open to what the LSP could offer and tried hanging on to old methods such as cramming.

### Teacher Professional Growth and Development

Professional development has been identified as one of the outcomes of DBR [23], and that has also been our experience. As the program was being implemented, the team learned from classroom practice, the literature, and professional colleagues. Conducting a semi-systematic literature review enabled an interdisciplinary team to learn extensively about learning development in the first year and to gain a common understanding. The manuscript writing process has also been a reflective learning opportunity, challenging us to rethink our practice, consider the literature and expand our understanding of regulatory learning.

As we have gained a better understanding of the supporting theories and practices, the components and teaching strategies of each LSP offering have evolved. For instance, it became clear that utilizing small-group learning was essential to support and socialize students towards learning in a PBL program. Through our reflections on the whole process, the importance and value of reflection for learning has become apparent. We have made room for explicit personal and group reflection in each session, after the first semester, and at the end of the year. Similar growth in our understanding has led to the inclusion of topics that support students’ development of other regulatory skills, such as goal setting and identifying learning gaps.

### Program Design Principles

#### Small groups

Although small-group learning is one of the core elements of PBL [22] at the beginning of medical school, most of our students prefer working alone and report being somewhat uncomfortable about learning in groups. Students have affirmed the benefits of collaborative small-group learning design for the LSP, benefits that seem to extend to PBL tutorials and other group-oriented learning opportunities. Over time, students have become more willing to join study groups and report improved interactions within groups; for example, being more likely to ask questions in small groups than in plenaries. Further developments in the program will leverage the emerging CoRL and SSRL literature to explore how we can integrate the understanding of student networks and regulatory processes [19] to foster improvement in PBL group learning.

#### Use of Reflection

Students may come to the learning environment with goals, beliefs, and strategies that are inconsistent with regulatory practice [17, 50]. LSP is designed to nurture reflection as a regulatory practice where students transform their experiences into learning [51]. In one of the reflection exercises, students reflect on the first year and write a letter to the upcoming medical students describing what worked well, what they wish they had known or done to be successful learners, and what should be avoided to “survive” the first year of medical school. Thus far, students’ insights have included the need for good time management, prioritizing learning, seeking help from peers and teachers, learning with others, and being open to learning new skills. Incoming LSP cohorts use the letters and other resources to reflect on and develop their strategy for success in the first year.

#### Using Biomedical Sciences Subject Matter to Facilitate Learning Strategies

Our LSP facilitates learning strategies using relevant biomedical science content, as suggested in previous work [20]. Students have affirmed the value of using relevant and current subject matter while learning strategies in LSP. Our students start anatomy in the second block (seventh week of the program), and almost all have reported challenges with conceptualizing anatomy. We think that students’ struggle with anatomy suggests the need for acquiring discipline-specific learning techniques.

#### Intervention Dose and Attendance

The benefits of a prolonged course duration have been documented in the literature [3, 20]. Our experience has been that the year-long course provides adequate time to address the necessary skills without burdening the students’ tight schedules. This presents a relaxed learning environment preferred by the students. Although the LSP is not compulsory, the attendance rate generally averaged about 75%, dropping to just over 50% for sessions held closer to an exam. It would be interesting to explore the learning experiences of students who regularly attend versus those who do not attend the LSP.

### Emergent Learning Success Program

The LSP aims to provide a supportive environment for students to develop the regulatory skills needed for self-management and independent learning. Table 2 summarizes the resulting LSP (key components, justification, related outcomes, and topics) developed by drawing from the literature, student feedback, learning from peers, and lessons from our own experiences. The use of the DBR approach has enabled us to add and modify elements and topics iteratively, and teaching approaches, e.g., renaming and restructuring the *Understanding yourself* to *A learner-centered orientation to learning* focusing on enabling students to confront and examine the conceptions of teaching and learning in relation to adjusting to PBL.

**Table 2:** Proposed LSP, including key components, justification, related outcomes, and topics

**Key component and description**	**Justification of component**	**Component outcomes and topics**
**A learner-centered orientation to learning** A conception of teaching and learning congruent with learner-centered practice, where the learner takes responsibility for their learning and views the teacher as a facilitator of learning and not the primary source of learning [9, 21]	Students' conceptions and practices may not be congruent with what is needed for effective learning in learner-centered environments [17, 21]. This component allows students to reflect on prior teaching and learning experiences compared to the current learner-centered environment requirements. The intent is to initiate conceptual change towards a learner-centered approach congruent with learning in PBL	**Learning outcome(s):** Reflect on prior learning experience and the requirements for the PBL curriculum and develop a statement of learning to guide learning moving forward **Topics:** Why the LSP? Types of transitions in medical school. Exploring prior and current learning contexts. Teacher-centered vs. learner-centered practice. The role of the learner in a PBL program. Competencies needed for effective learning. Tips for surviving a PBL curriculum. Preparing the learning environment
**Academic and professional skills** A toolkit of learning skills and strategies, including information literacy [resource location, appraisal, and management] [9, 52] driven by process of determining learning goals (forethought), executing them (performance), and evaluating one’s methods and progress (reflection) [15]	Students need a repertoire of cognitive, motivational, and behavioral strategies to manage learning tasks [38]. However, they may not bring the necessary skills needed for academic success to the learning environment. Poorly achieving students may have a limited toolkit of learning strategies [53]. Some may go through schooling having not acquired effective and efficient learning strategies [53, 54]. As such, we cannot assume that students can self-regulate their learning when they enter medical school [6, 55]. This component is intended to provide learners with a repertoire of all three types of strategies [38] to choose from when completing learning tasks and skills to regulate their learning [15]	**Learning outcome(s):** Use learning and study strategies for effective learning in a learner-centered environment. Develop and deliver effective presentations and display a professional demeanor during presentations **Topics:** Understanding your brain (diffused and focused modes of thinking). Types of learning strategies (rehearsal, elaboration, organizational). Learning strategies (minute paper, concept maps/mind maps, muddy points, teach-and-learn, focused list, memory matrix, *SQ3R, **KWL chart). The study cycle. Using block guides as a learning tool (learning outcomes, Miller’s pyramid/Bloom’s taxonomy). Test-taking strategies. Tips for learning anatomy. Presentation and poster design. SRL components: (forethought, performance, reflection)
**Collaborative learning** Collaborating effectively with peers and utilizing them as a resource for learning [9], including skills associated with understanding others, working effectively in teams, leading teams, managing team projects, and evaluating self and others [52]	Most students enter medical school reluctant to participate in groups and may display ineffective group function behaviors. However, medicine is a team-oriented profession where effective collaboration is essential for safe, high-quality, patient-centered “care” [12]. Additionally, small group learning is the core of learning in a PBL curriculum. This component is intended to draw from regulatory learning theory [17] (SRL, CoRL, and SSRL) to socialize students toward collaborative learning [18, 19]	**Learning outcome(s):** Display professional collaborative behavior when working with others **Topics:** Learner differences. Tuckman’s group function model. Listening. Giving and receiving feedback (feedback literacy). SSRL/CoRL. Interacting well with others both socially and professionally. Working towards a common shared goal. Building meaningful relationships. Empathy (understanding others’ feelings and responding appropriately)
**Professionalism and ethics** Skills related to professional and ethical behavior, including culturally oriented elements of professionalism	Professionalism issues during students' medical training can carry into their practice as graduates if not explicitly addressed during education and training [56]. This component sensitizes students to elements of professionalism, especially drawing out those derived from our cultural context	**Learning outcome(s):** Summarize the expected professional standards for doctors and medical students and the roles played by regulatory bodies **Topics:** Four principles of bioethics Professional demeanor (*Botho*). Building a professional brand
**Self-management** Use of skills and strategies for managing oneself, the learning environment, and the external environment that can otherwise affect learning	Non-academic factors, e.g., psycho-social and socio-economic, can affect learning. As such, effective learning requires regulation not only of cognition but also behavior and affect [17, 38, 57]. This component provides students with the personal management skills needed for effective learning	**Learning outcome(s):** Use effective skills and strategies to manage self and the learning environment **Topics:** Self-awareness. Self-care (stress management, work-life balance). Goal setting (****SMART technique). Time management (delayed gratification and procrastination). Emotional intelligence. Financial literacy. Resilience. Motivation. Help-seeking. SRL components: (forethought, performance, reflection)

*Survey Question Read Recite Review; **What you know, what you want to know, what you have learned; ***Setswana expression for respect, good manners, and good character, ****Specific Measurable Achievable Relevant and Time-bound

#### Study Limitations

While there are some insights new medical schools may glean from our experience, especially those from low-resourced settings, our findings are limited to our students and our context. When writing the manuscript, we had only completed 2 years of program intervention and observation. Also, our students are undergraduate students and may not have the learning experience of graduate medical students. Student data were analyzed and interpreted by insider researchers with vested interest to see the program succeed. Although largely a positive in DBR, this does risk some inherent subjectivity, which we acknowledge, and have tried to balance through consultation with external colleagues.

#### The Way Forward

To date, the development of the LSP has focused on identifying its components and gaining a better understanding of its theoretical grounding. There is a need to develop a more robust evaluation strategy utilizing a mixed-methods approach [25, 58] to assess how students respond to various components [24] and determine appropriate outcome measures for evaluating the LSP. As we gain experience with other forms of regulatory learning, future development should explore explicit exposure to CoRL and SSRL skills and strategies [18, 19]. Furthermore, it is essential to extend professional development to the teachers to integrate regulatory learning as part of biomedical sciences and clinical skills teaching. This can enable teachers to explicitly model and teach co-regulation strategies in tutorial groups to enhance group function and learning [17] and build capacity to complement the LSP with a remediation process for at-risk students. Now that we have a preparatory program that is starting to mature, there is a great opportunity to provide remediation support to failing students.

## Conclusion

The incremental nature of DBR has allowed for developing a context-specific program that considers students’ voices through needs assessment and feedback on the program offerings. Overall, our students describe the LSP as adding value by enabling the adoption of a repertoire of skills and strategies for learning management. Working in small groups has provided opportunities for students to become more comfortable, learn, test their thinking, and establish their voice among peers. DBR has also provided an opportunity for the professional development of teachers through feedback from classroom practice, reflection, and the literature.

## Supplementary Information

Below is the link to the electronic supplementary material.

Supplementary file1 (DOCX 434 KB)

## Data Availability

Data can be found in the supplement included with the manuscript.
