# Supplementary material for: Design of a Learning Development Program to Support First-Year Undergraduate Medical Students in the Transition to a PBL Curriculum
Source: Med Sci Educ. 2023 May 4;33(3):755–65. doi: 10.1007/s40670-023-01790-3 (PMC10368596; doi:10.1007/s40670-023-01790-3)
Supplement: Supplementary file 1 — Supplementary file1 (DOCX 434 KB) [file 40670_2023_1790_MOESM1_ESM.docx]

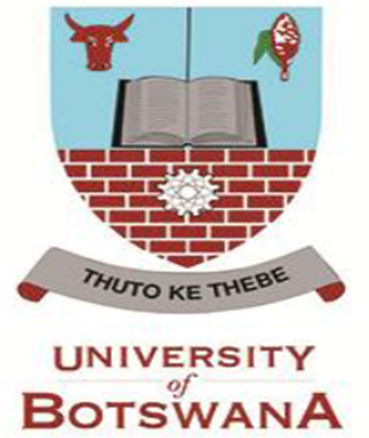


**Masego B. Kebaetse**

Coordinator and Co-facilitator

Department of Medical Education, Faculty of Medicine

**Brigid Conteh**

Co-facilitator

Communication and Study Skills Unit, Centre for Academic Development

FACULTY OF MEDICINE

**MBBS Year 1 Learning Success Program**

**2018-19 Academic Year Report**

**Created:** July 2019

**Revised:** March 2021

Contents

[Background 3](#_Toc130405349)

[Phase 1: Needs Assessment 4](#_Toc130405350)

[Phase 2: Design and Delivery of the LSP Pilot (2016-17) 15](#_Toc130405351)

[Feedback on the 2016-17 Semester-long Learning Success Program 15](#_Toc130405352)

[Summary of Findings 16](#_Toc130405353)

[Phase 3: Iterative Cycles of Delivery and Refinement 24](#_Toc130405354)

[The 2017-18 Semester-long Learning Success Program 24](#_Toc130405355)

[Feedback on the 2017-18 Semester-long Learning Success Program 26](#_Toc130405356)

[Summary of Findings 27](#_Toc130405357)

[The 2018-19 Year-long LSP 33](#_Toc130405358)

[Feedback on the 2018-19 offering of the Learning Success Program 34](#_Toc130405359)

[Summary of Findings 35](#_Toc130405360)

[Reflections and Observations 41](#_Toc130405361)

[Conclusions 43](#_Toc130405362)

[References 44](#_Toc130405363)

# Background

The Learning Success Program (LSP) was developed in response to a disquiet that some of the teaching staff in the Faculty of Medicine (FoM) had regarding first-year medical students' perceived poor adjustment to the Bachelor of Medicine Bachelor of Science (MBBS) problem-based learning (PBL) curriculum. During the forming years of the FoM's, first- and second-year medical students had to submit a written reflection that synthesized and integrated their week's learning across disciplines, learning events, and experiences. Some faculty members were dissatisfied with the poor integration of concepts and lack of depth when responding to questions requiring higher-order thinking. The initial explanation faculty members ascribed to the observed situation was that the students were experiencing writing challenges. However, students were also observed as exhibiting "unproductive" behaviors, such as shortcutting the brainstorming process, rushing through PBL tutorials, not asking questions, not challenging one another's thinking, and displaying a competitive attitude that stifled the open discussion needed for learning in the PBL tutorials. Upon further reflection and literature review, the team considered student challenges beyond writing. The team concluded that there was a need for a learning development program, herein referred to as the Learning Success Program (LSP), to support students' adjustment during the first year. Subsequently, a decision was made to conduct a systematic investigation to explore first-year medical students' learning experiences during the first semester of a PBL program.

# Phase 1: Needs Assessment

To understand our students' specific challenges, the research team, comprising six academic staff, two educationists, and a medical student, was assembled. The team conducted a qualitative study (needs assessment) to explore learning challenges experienced by first-year medical students during the transition to a PBL-based program, a learning approach that students had not experienced in their prior learning. Fifty first-year medical students and their six PBL facilitators were purposively sampled ^1,2^ to participate in the study. Twenty-three first-year students and five PBL facilitators (three of whom are study researchers) agreed to participate in the study and stayed on throughout the study.

Data were collected through in-depth interviews and non-participant observations. Interviews generally lasted 90 minutes, were audio-recorded, transcribed verbatim, and the transcripts reviewed against the audio to ensure accuracy. Drawing from the constructivist grounded theory data analysis approaches,^3,4^ we analyzed data using thematic analysis^5^ in search of patterns in the data. This included familiarization with data, open coding^10^ to generate initial codes and categories, searching for, defining and naming themes, reviewing and consolidating the themes, and determining relationships been themes.

In light of our medical school's newness and some faculty members' newness to university teaching and/or teaching in a PBL program, the research team also familiarized itself with related literature throughout the study. The study's findings (Table 1) provided the necessary information pointing to challenges students were experiencing in the first semester. The findings and the literature were used to develop an intervention to support student learning in the first year.

| **Table 1 Summary of themes identified from the qualitative data** | | |
| --- | --- | --- |
| **Theme** | **Codes** | **Data excerpts** |
| Students experience a range of learning challenges during the transition to the PBL process | **Students' perspective on challenges**   - Heavy workload, program is stressful - Uncertainty about required breadth and depth - Need to assume greater responsibility for learning - More learning effort needed in MBBS: - Difficulty adjusting to a new environment. It takes about a semester to get the lay of PBL. - Limited understanding and application of the PBL process | **Heavy workload -** G2S4: The amount of material we covered in 201, ah, it amounts to the whole of BSc [laughing]. I tell you, that was the whole of BSc. G1S5: It's really stressful. G2S1: I was stressed out. I was really stressed out. G3S2: … a lot of pressure. G2S3: I was too pressured. Actually, at first I was so freaked.  **Uncertainty about required breadth and depth -** G1S4: Sometimes…my classmates would know a lot…they’ve gone in depth and you’re on the surface. Or sometimes you feel that you’ve gone into depth and others are just on the surface. G1S8: You don’t really know how deep you should go. Most of the time we are too deep…way ahead of ourselves.  **Need to assume greater responsibility for learning -** G3S2: So here, you have to self-motivate […]. You don't get motivation from your lecturers […]. So, you get to have self-responsibility, drive your own things, make your own life timetable, know what to do at what time […]. I just learned, like, self-responsibility. G2S8: I know there is that distance between senior school, and, um, BSc where you're not really spoon-fed as much. But you kind of are [spoon-fed] in BSc. They give you everything, examples and whatnot. All you have to do is just study on what they taught you. *But here, you have to really get hands-on and study and make sense out of stuff, and they teach you a portion and you have to just do the rest. It's more of self-learning*  **More learning effort needed in MBBS:** G1S5: Yeah, I didn't study a lot. G2S1: Okay, BSc, what used to happen is that you find that during the day, 'cause with BSc, unlike here, we even had classes in the afternoon. .... Then what I would do with BSc is I would just chill. Go talk to friends. Do something. Then, between that 6 and 11, you find that I would study from maybe 8 to 10 and that was tomorrow's class, every day I didn't have to study frequently at all. I wasn't really much of a person who studied frequently.  **Difficulty adjusting to a new learning environment -** G1S9: For the first block we were just new. We were not yet comfortable. G3S8: First of all, it's just, um, words and then the different aspects of, like, embryology and histology, and physiology, all that. Not really the content but it's just, it's just different. G2S1: Yeah, I really think I've learnt it, and I don't have much problems with either as when I had started. 'Cause at first I was like, "Ah, no! What is this now?" Yeah. I was, you know how, at first, I was isolating it from the plenaries. It was more like, there's the plenaries, there's PBL there. F1: You'll find that it's hectic, not just the volume of work, but also it's PBL, something they have never, almost everybody, if not everybody who comes here as a student, has never experienced it. G2S2: I thought B.Sc. was difficult. B.Sc. is water. It’s just simple. B.Sc. is simple. The thing is, with this course, you can’t afford to miss a plenary. One, content, the volume of content is just too much. It’s no longer about how smart you are. The level of intelligence got you here, but the work you put in makes you stay here. |
|  | **Faculty perspective on challenges**   - New learning environment - Undermining the PBL process - Difficulty judging depth - Poor integration - Poor engagement | **New learning environment -** F1: You'll find that it's hectic, not just the volume of work, but also it's PBL, something they have never, almost everybody, if not everybody who comes here as a student, has never experienced it.  **Undermining the PBL process -** F3: And they seem to be trying to finish quickly to skip the process. F3: They seem to be… to now to be deciding almost in advance what the learning objectives are going to be. They're not… they're brainstorming the cases and then ignoring the brainstorming and they… and just writing objectives and then not really researching the objectives properly sometimes. F2: The students, or the group, one of them, or two of them, or a couple of them in the group actually circumvent the, or short circuit, that’s the word I want to use, the brainstorming. […] but if you short circuit the brainstorming, you are not going to go anywhere because you have not actually, you know, used that incorrect knowledge.  **Difficulty judging depth - F3:** They don’t seem to be understanding the depth that they need to go to, skating over the surface.  **Poor integration -** F6 - [..]The group as a whole, the first years, they have little problem in putting it together.  **Poor engagement** – F1: Vigorous engagement, or engaging of one another, is something that I think is a weakness. They improved since they started, but I'd say, it's something that definitely I would like to see more of. |
| The attitude towards PBL is influeced by the understanding of the PBL process | - Uncertain about PBL and its value in the beginning - Initially don’t see the case but the plenaries as the driver for learning - Some understand the role of the case early on - Challenges with brainstorming - Confusing brainstorming for diagnosis and trying to solve the patient’s problem - Not challenging each other’s thinking | **Uncertain about PBL and its value in the beginning** - G1S6: At first I hated PBL….I didn’t understand the concepts. I started to get the importance of PBL in 202 and 203 (the first two system based blocks) I loved it. G1S4: At first when it started I thought it was a waste of time…..now after doing it I realise that it is actually important….a way of learning and studying.  **Initially don’t see the case but the plenaries as the driver for learning** - G2S3: They (PBL cases) kind of support what was taught in the lectures. G1S3: (In PBL) we cover what we have already covered in the plenaries (lectures). It's just revision.  **Some understand the role of the case early on -** G2S8: It (PBL) does link with the stuff in plenaries but you’re going to meet cases and you’re going to have to research about them….and that’s how you’re going to be learning. G2S2: I know that if I do my PBL thoroughly I’ve covered everything that I’ve learned this week. So I use PBL for studying.  **Challenges with brainstorming -** G1S2: Most difficult? I’d say its brainstorming. That’s the challenging part where I feel we get stuck most. G3S1: At the end of the semester we still have difficulty knowing exactly (how we should do) the brainstorming. G1S8: We have done this for three blocks and still don’t know what brainstorming is….you’re hypothesizing …. about the solution to the problem.  **Confusing brainstorming for diagnosis and trying to solve the patient’s problem -** G3S2: Initially we thought of having to find treatment for that patient. F5: They want to learn about disease…to be a doctor. I am telling them go back to basics….after the second, third or fourth time they understand. F2: We are emphasizing basic science in year 1. We don’t want them to bother about diagnosis…management….prognosis.  **Not challenging each other’s thinking:** G3S8 - Everybody is just reading what they have and the minute I just break and breathe, the next person has already started reading and nothing was said about what I said. And no comment whatsoever. And people, um, people rarely correct each other even though they do mistakes, they rarely do. Our facilitator would be like, "No, did you really hear what he said? It's not correct. Why didn't anybody say anything?" And I think it's because people are just rushing to say something. Or they don't really listen to, they are like, "Ah, just begin already" [laughing]. |
| Students who adopt self- and social-regulatory study strategies feel more successful and less stressed | Place of study | **Place of study -** G1S2: I prefer studying in my room ... it’s quieter. G1S3 I usually use the library or 247. [Laughs] Just because it’s conducive. It is not as hot compared to the rooms. But there’s nothing much different from the library, my room, my room is also okay for study. G1S7: Then I’ll study from then till 5 [laughing] in my room. I do live on campus, I stay on campus. G2S1: The times that I study in the room, I’d say, it’s when I have to study late night, late at night. That’s the only time I use the room. But basically, from after classes, it is the library. If I have to study during the day, it’s the library. If I have to study in the evening, it’s the library. |
|  | Study times | **Study times -** G1S3: I find it hard to study in the afternoon. G1S7: I sleep around 7 or 8, then I’ll wake up at 12 midnight [giggling]. Then I’ll study from then till 5 [laughing] in my room. 12 midnight, that’s when I’ll start studying. Like I’ll read, seriously reading. Then from there, maybe at around 4, I will just recap, what I was studying from 12 to 4. |
|  | A variety of study strategies | **Writing notes:** G2S1: Um, briefly, I write short notes, really. Not out everything that is, I write short notes of those concepts. If I read a concept through, then I realize, “Oh, I think I really understand this”, I don’t really put it down. Or I just put down a little, little, little. But then, I usually in the process, in my studying process, I almost all the time I write. G1S9: I think that’s my basic way of learning. I write notes, short notes for myself trying to summarize what I’m reading. [...] And after writing my notes, and then the next time when I just look at them, it becomes as if now, yeah, it becomes easier. Like right now, I’m studying for the exams, I’m using my notes, so the moment I just look at what I have written, it just sticks right, just like that.  **Drawing and mind maps:** G3S2: I do draw pictures sometimes, mind maps and all that. Yeah. G1S2: I can’t learn without writing stuff down. Then diagrams, basically, put a diagram on a slide, then I will just draw that and tie the sketch to it. That’s the only I use. G1S8: If you’re drawing and someone looks at you, saying that, “What’s she actually doing? It’s like playing with pens.” But, it’s actually fun and helpful.  **Use of videos:** G1S4: The videos, definitely. They simplify the material and then when you go to the book, you have an idea of what, what you’re reading. [...] I’ll get all the books that I need, and maybe use the internet, and then look for videos that I could watch, to simplify before maybe using the books. Then use the books, and then look at the lecture notes. G1S3: I also sometimes use YouTube, those kinds of things, the videos[...]. More especially when I think, “Eish, I’m tired from reading the notebook”, I will just stick to videos because… [laughs]. The picture gives me a better understanding as compared to reading something that is written. G1S5: Well, I use books. I read, write, and then from the supplementary videos from the internet. G3S4: It can appear really hard. So you use the video to see the animation there.  **Working alone:** G1S5: Groups? Lately it hasn’t been working. I’m very much of a loner. I enjoy studying alone. G2S1: I’d say from high school, even through BSc, I employed the same style of learning, I’d say. ‘Cause with me it was just going to the plenary, then after that, taking my textbook, notebook, putting it there and studying on my own. I always did alone. G1S9: I don’t know, I guess as well, they are as well accustomed to studying alone. So, having to get people together to study, to study in groups, it’s really a hassle. Nobody really wants to do it. But I’ve tried it once and it kind of worked as long as you’re with cooperative people. Yeah, it really works. But then finding people to actually do a group study together, it’s not easy.  **Working in groups:** G2S1: I tried it once, I remember with anatomy, with my group mates, we booked a lab and we sat around the table and we discussed. And surprisingly, it was much more effective than me there being alone trying to gather around. G2S2: I believe that working in groups, we cover a lot because you bring different people who contribute different aspects and that for me in the past has worked. But coming here to university, it was a different agenda. Everybody is pushing their own agenda up in here but I tried formulating a group, but I feel to a certain extent people are just too competitive and I haven’t found those compatible people that, um, were not about the competition or who’s going to get more or what. We’re just here to help each other. I know something that you don’t know and you know something that I don’t know so if we could work together and not hold back. G1S3: Even the small group or the group that I am involved in is helping me because the more I share or exchange information, something like that, the more I get to know something. It’s very hard for me to forget it. Even the anatomy, anatomy labs they helped a lot. G2S1: I tried it once, I remember with anatomy, with my group mates, we booked a lab and we sat around the table and we discussed. And surprisingly, it was much more effective than me there being alone trying to gather around. |
| Some students see the benefit of working in groups, while others prefer working alone | - Benefits of learning in a group - Positive group function - Negative group function - Silence | **Benefits of of learning in a group**  G3S1: The fact that we will just be there together sharing ideas, it's actually nice because somebody comes up with something you didn't find so you get to hear about it and you exchange ideas. And yeah, if something you didn't understand, somebody explains it much better than a lecturer, not exactly like a lecturer, but it's easier. You hear it from a student like you than from a lecturer. So it's actually nice. ... Yeah. And it's easy to ask a question to your age mate because you'll just be few in a group. So, it's really easy to ask, "what do you mean by that?" Yeah.  S102: Well, I think it's just a matter of, because, I think it depends on what, like, the topic. If I go to class, okay, fine, we are doing about this thing. And, I know I don't understand much on it, I will always be like, "fine, hopefully somebody will come up with something that will make me understand". And I will listen much more than something that I really know. So, I'll just be like, "okay, fine, I know that". Yeah. But if it is something I don't understand, I will pay attention and try and get some sense.  G2S4: Yeah, sometimes, yes, sometimes, no. Sometimes, I feel, no, ah, uh, this one had already been covered through our lectures. What someone said is exactly what they took from the lectures. And sometimes you feel, "Ah! Yes, I learnt a lot today". Mm.  **Positive group function**  G3S8: [I] like the groups I've been in since our PBL and clinical placement, we're quite okay, as in, I can't imagine, we're okay, we're good, we're all chilled people. So we just discuss and everybody is just okay. But then I hear the other groups who are, like, always attacking each other, and I'm, like, okay [laughing]. ... Yeah, other PBL groups. Ours is just comfortable. It's really good. Ours is very good. I don't know what's going to happen when they swap us around [laughing]. But, yeah, I don't mind group work. Group work works a lot actually. Yeah.  **Negative group function**  **G2S2:** Everybody is pushing their own agenda up in here but I tried formulating a group, but I feel to a certain extent people are just too competitive. **G3S1:** Most of the time, we don't listen to each other. […] it's more of me saying what I have to score marks. […] I think that's all it is to it and you don't actually listen together. Because, even if you make a mistake or you say something that is not right, people can't realize it. **G3S8:** But, and most people, like, with the whole competitive issue, they don't even care about what you said. They just want to say what they have. **G3S1:** Because sometimes there are people who like overpowering us. They say a lot and then they say everything that, even those things you have found out. So you'll actually be like, "ag, somebody will say it out. I'm not doing anything today".  **Silence**  F1: So that was the weakness, some students were quiet, you know, generally on the quieter side and needed to be nudged, um, and I had to do it. F3: It can sometimes be like drawing teeth trying to get people to talk in PBL groups. G1S4: It [silence]has improved very much. I think, then we were not sure how exactly we were supposed to present PBL. It was about being confused and not being sure of what exactly is expected of you. Or what you're to do. So, with time we adjusted. G3S8: In our group, in PBL, in our group, um, when we have that Thursday session and everybody is, well, people just nod really or they just keep quiet. They don't really, I don't think that's, I would prefer if they'd say something or comment on what I said. |
| Students are uncertain about which resource to use and how best to appraise them | - Use of textbooks - Use of the internet - Use of medical sites and apps - Judging the credibility of internet sources - Trusting information from peers - Need to interrogate multiple sources | **Use of textbooks -** G1S7: Mostly I use my textbooks and slides, but mostly slides. Cause they do set from the slides, the lecturers. G3S4: I am using the textbook, the slides, I only take a small amount of information from the slides and a lot from the textbook. G3S5: Normally I use textbooks. Yeah, I don’t like the net. Yeah, because net, ah. I do use it, I do sometimes get in Google. G1S6: But I sometimes feel it’s helpful to read the textbook because, if you read the textbook, you’ll be prepared for both the, I think you’ll be preparing for both the tests, or the plenary and the PBL. F306: But I think that they can get a lot of what they need to know from textbooks. Sometimes, and I don’t think they necessarily use the right textbooks. I see them, for instance, trying to get everything from, say, the physiology textbook.  **Use of the internet -** G1S6: Most of us, we get it from the Internet. Because our facilitator even, he wants us, asks about, “Where did you get that? Where did you…”. And everyone was like, “Internet”. I think only one or two said from the books. Yeah. G2S1: It’s random. But mostly I think I use the My Clinic website because it helps out a lot. It usually has information more especially pertaining to our cases. I mean it’s rare for me not to find anything on it. But I think, most of the time, it’s really randomized.  **Use of medical sites and apps –** G2S3: Yes. I use my textbooks and there are some medical websites, like Up-to-date, Med Link, and all those I use to, I download. G2S5: I use some medical, the, these useful medical sites, Bio-clinic.  **Judging credibility of internet sources –** G1S9: 'Cause you don’t really know how, if they’re really reputable. Or you just come, you get information because information is provided there and it looks concrete. You just put it down.  **Trusting information from peers -** G1S2: Only talk about references when I don’t trust my colleague’s source.  **Need to interrogate multiple sources -** F5: And they have to do something deep in research also not like sitting and reading one book, it’s not, they had to go for a variety of books and do research. |
| Many students failed to appreciate and or utilize the support available to them by the university, often turning to other sources for support, but acknowledge the need for a dedicated program with the medical school | - Students' views on university support - Negative attitude towards COM - Upper-class students as a source of support - PBL tutorial as a source of support - Psychosocial support from family - Acknowledgement of struggle with transition - Facilitators help students with a way to frame their thinking - Views on the need for a learning support program | **Students' views on university support -** G2S3: I won't say there was much support from the university. G3S4: No learning support in SoM. G3S8: Okay, not in the School of Medicine. G2S1: I am not attentive to the initiatives set in place to help us learn, … maybe some are provided. G1S3: Support is there, it's just I haven't went to seek one. But the support is there.  **Negative attitude towards COM** - G2S5: … we hated COM. Honestly, we did not want COM. G3S3: Didn't learn much from it because I had an attitude towards it. G1S4: I really thought COM was irrelevant... G2S3: We didn't do much in COM. ... we used to disregard it. We really didn't take it seriously. So I think that is why I can't even think of COM. G1S5: I never took COM seriously. I thought it was boring. Yeah, I did attend class every time.  **Upper-class students as a source of support -** G2S3: I like to consult with our fellow colleagues who are ahead of us. G1S9: ... from people who have got experience about the MBBS, who have gone through the MBBS before. G2S2: …point us in the right direction of where the tests are headed, because in this school there is no such thing as revision papers. G2S6: I think people who came across the same challenges, they can really help you about it. G2S2: So, a person better suited to advise you, except for the lecturers, are those ahead of you.  **PBL tutorial as a source of support -** G1S7…the PBL sessions, I think yeah, they are kind of supportive to our learning. G2S5: He told us we have to think anatomy first, .... this is how this thing works. G1S4: The facilitator would say 'think about the anatomy, think about the physiology, think about the public health'…..it helped. It triggered the transition. G1S7: Our facilitator once told us that we should come prepared. So I think it kinda helped.  **Psychosocial support from family -** G3S1: Most of the time she [mom] is the one who encourages me. […] after I wrote an exam and I feel it was very difficult, I call her crying, and then she just comforts me. G2S1: I talk to my parents almost every day about my schoolwork. And, yes, they try to encourage me. They try to find ways of, if at all they can do something about it, they do something about it. Yeah. So, I do get a lot of support from my parents.  **Acknowledgement of struggle with transition:** G3S3: I think I'm still struggling... that transition, you know. I have to study a lot, read a lot, and carry my books around a lot. I think ... if I was taught how to handle all that, I think that would help. G1S6: ...for the first block we are really struggling even for the PBL. G1S3 ... it will be of assistance, looking at the way most of my colleagues' approach or study. … I don't mean to be judgmental, but I don't think it's quite effective. G1S4 ... we do adjust in the long run, but the thing is, it takes a while for others. Some take a shorter time to adjust. So, if there was somebody to help us, maybe, you'd adjust quickly.  **Facilitators help students with a way to frame their thinking -** F6: They start with anatomy, the physiology, then to pharmacology, maybe microbiology or parasitology then to public health. G1S4: The facilitator would say ‘think about the anatomy, think about the physiology, think about the public health’…..it helped. It triggered the transition. F1: I share with students all the time. I really think that it is quite important, um, that students follow a certain method. And method would be something along these lines, um, attend all lectures, sessions such as PBL, um, labs, take advantage of all the interactions that the faculty have with you as a student. And then, when things are still fresh in your mind, whether you have challenges or not, that evening, take a quick review at your notes. Once you think that you have put in quality time, done your due diligence as far as studying, get together with a group that you can work with, a study group, where, as I say to students, um, you review the material together, and get to a point where you feel like you have a good understanding or grasp of the subject matter.  **Views on the need for a learning support program -** G2S1: Helpful, G1S7: vital, G1S8: important, G1S6: especially for first years. G2S7: … this [MBBS] is a very stressful course. You need some skills, you know, serious skills. |

# Phase 2: Design and Delivery of the LSP Pilot (2016-17)

The LSP was piloted as a semester-long program in the 2016-17 academic year. It was intended to equip first-year medical students with the necessary learning skills for succeeding during their transition into the PBL curriculum. For the 2016-17 academic year, the program focused on the learner's roles and responsibilities in a learner-centered PBL curriculum as an essential element of self-directed learning. The needs assessment findings informed the program outcomes and topics (Table 2).

| **Table 2: Summary of outcomes and topics included in the 2016-17 Learning Success Program** | | |
| --- | --- | --- |
| **Primary outcome** | **Learning Outcomes** | **Topics** |
| Develop and implement a strategy to excel in a learner-centered environment | Articulate the role of a student in a learner-centered environment | - Types of transition in medical school - What is learning, eight things needed to be an effective learner^6^ - Teacher-centered vs learner-centered teaching, exploring prior and current learning contexts - Learning preferences and implications for learning |
|  | Use effective study strategies for a learner-centered environment | - Learning strategies: minute paper, concept maps/ mind maps, teach and learn, focused list, memory matrix - Self-management strategies: time management |

### Feedback on the 2016-17 Semester-long Learning Success Program

*Data collection:*

At the end of the semester Learning Success Program in September 2016, the workshop facilitator sought feedback from students to improve the design of the 2017-18 LSW offering. Students who were willing to participate were invited to provide feedback on the LSW. The feedback session was not facilitated by another faculty member from the Department of Medical Education instead of the workshop facilitators to reduce coercion. Data were collected through written reflections using several open-ended questions:

- What is the most significant learning? What did I learn?
- What is still unclear? What am I anxious about? What would I still like to learn?

*Data analysis:*

Thematic analysis^5^ was adopted to search for patterns in the data.^3,4^ It included familiarization with data, open coding^10^ to generate initial codes and categories, searching for, defining and naming themes, reviewing and consolidating the themes, and determining relationships between themes. The themes identified from the data were summarised as shown in Table 3 and Table 4 below.

### Summary of Findings

*What is the most significant learning? What did I learn?*

Regarding students' perception of significant learning experiences during the program, students reported increased awareness of their role as learners in a PBL curriculum, having learned the value of group work, including getting the value added by learning with and from their peers. They also reported increased awareness of and benefits of having a toolkit of various learning and study methods. Additionally, they reported awareness of learning approaches for medical school, including integrating information, information literacy, the need to be actively engaged with learning, effective time management and self-organisation, and the need to think like a doctor, such as demonstrating critical thinking and applying content to clinical situations.

| **Table 3 Themes related to significant learning during the Learning Success Program** | | |
| --- | --- | --- |
| **Theme** | **Codes** | **Data excerpts** |
| Students recognize that their role in a PBL program requires more self-direction - changes in the use of strategies and assuming more responsibilities for their learning | - Reflection on prior learning as teacher centered | Most of my learning was centered on the teacher, but now I get the sense that most of the learning is going to be done by me [LSW-16-SL-15] |
|  | - Open to the need for a change in learning strategy | Be open to learning at all times [LSW-16-SL-37]. I recognise the need to change my learning methods from the BSc to a more learner-centered approach which is in line with the PBL method [LSW-16-SL-35]. I learnt that the method of learning in MBBS is different. Therefore, I need to change my approach in terms of how I learn [LSW-16-SL-36]. |
|  | - Overwhelmed with a lot of information | During the session, it made me reflect on my transition from secondary to university, and I realise that I am overwhelmed with a lot of information [LSW-16-SL-49]. |
|  | - Gaining insight into the role of PBL and the PBL process | I have learnt that the plenaries are just an overview of what we have to learn and that I have to go out and look for the information myself [LSW-16-SL-30]. The majority of the learning will be self-learning through PBL [LSW-16-SL-12]. PBL requires the learner to be able to use the knowledge acquired to real-life situations [LSW-16-SL-42]. I also have to take PBL sessions very seriously [LSW-16-SL-30]. I got to appreciate why we are having PBL sessions to apply the theme of the week to real-life situations [LSW-16-SL-34]. |
|  | - Increased learning effort | I have learnt that I should do more work [LSW-16-SL-44]. I have to improve on my learning approach by reading more and researching on my own [LSW-16-SL-48]. |
|  | - Learning at SOM as a learner-centred environment | I learnt that in order to survive in medical school, I have to use a learner-centred approach [LSW-16-SL-47]. It is important to understand that the way we learn is different, it is more learner-centred and thus, we should be able to learn on our own [LSW-16-SL-26]. One should not only rely on lecturers but should read other information sources [LSW-16-SL-44]. I learned about student-centred learning that emphasises independent learning [LSW-16-SL-42]. |
|  | - Increased awareness of the role of self-directed learning | The most profound thing I learnt is that I have to be a self-directed learner and work harder. I need to change of how I perceive my studies and be more time-conscious [LSW-16-SL-46]. Design my own way of studying or self-directed learning [LSW-16-SL-06]. Self-directed learning and limited time [LSW-16-SL-19]. I learned that self-learning is very critical in FOM [LSW-16-SL-10]. I realised that if I do most of the learning myself, I will learn better rather than depending on the lecturers [LSW-16-SL-20]. What I got to realise today is that in Medical School, we have to learn on our own [LSW-16-SL-11]. |
|  | - Confidence in ability to direct self-learning | I learnt to believe in myself to do more and do more self-learning as opposed to depending on the lecturer [LSW-16-SL-22]. I realised that if I do most of the learning myself, I will learn better rather than depending on the lecturers. I will be able to recall information and use it critically and integrating the information [LSW-16-SL-20]. |
|  | - A personalised learning strategy | I learnt that there is no pure approach to learning. The key is to identify what works best for you as an individual [LSW-16-SL-40]. Today I learnt that in UB FOM an individual should be able to device a learning strategy for themselves because Learning in UB SOM goes beyond the classroom [LSW-16-SL-14]. Understanding the learning approaches adopted at SOM [LSW-16-SL-05]. The most significant thing I learnt today is I need to learn how to study effectively in Medicine [LSW-16-SL-18]. The reflective exercises made me realise how I have progressed in terms of how I am learning and how I have improved at each stage [LSW-16-SL-29]. I learned on how to make a transition for learning [LSW-16-SL-01]. I have learnt that learning is supposed to be strategic and well thought of [LSW-16-SL-03]. The learner should be in a position to know and establish how much they should learn with guidance from the lecturers [LSW-16-SL-42]. How to come with the best approach to learning for PBL [LSW-16-SL-21]. |
| Some students see the benefit of working in groups | - Healthy interaction with peers | Healthy ways of interacting with my peers to enable a better learning environment [LSW-16-SL-01]. Learnt how to interact well with others [LSW-16-SL-04]. Also, the importance of respect for others, listening and communication are very important [LSW-16-SL-39]. |
|  | - Collaborating to learn from each other | We have to learn to collaborate with others and merge what we know [LSW-16-SL-37]. |
| Students Recognize the need to adopt a variety of approaches for effective learning at SoM | - Thinking like a doctor [acquiring critical thinking, application of content to the clinical situation] | **Using approaches that work for learning at SoM**   - **Thinking like a doctor [acquiring critical thinking, application of content to the clinical situation]:** To learn how to be a doctor, you should be able to integrate information and use it to solve problems [LSW-16-SL-43]. I have learnt that I need to acquire critical thinking skills in order to be able to think like a doctor [LSW-16-SL-01]. That I should apply my problem-solving skills in real-life situations [LSW-16-SL-06]. |
|  | - Active and engaged learning [engaging with learning for better outcomes] | - **Active and engaged learning [engaging with learning for better outcomes]:** Most significant thing I learnt was that the person who is doing most of the work does the most learning [LSW-16-SL-08] [LSW-16-SL-09] [LSW-16-SL-25] [LSW-16-SL-43] [LSW-16-SL-25] [LSW-16-SL-33] [LSW-16-SL-06] [LSW-16-SL-06]. |
|  | - Information literacy [looking for relevant information] - Integrating information | - **Information literacy [looking for relevant information]:** I should improve the way I locate information [LSW-16-SL-48]. We should also learn how to look for information [LSW-16-SL-42]. I learnt that learning how to locate information is a skill that I need to acquire [LSW-16-SL-40]. I have to believe in my analytical skills and find the right sources of information [LSW-16-SL-23]. I should look for relevant information to solve problems [LSW-16-SL-44] |
|  | Application of knowledge to other contexts | What we learnt in anatomy can be applied for a clinical question [LSW-16-SL-24]. Learning is not only restarting what is in the textbook but be able to relate topics when faced with real-life problems [LSW-16-SL-43]. Learning at the faculty of medicine is learner-centred. Learning is putting into action the knowledge gained. The learner-centred approach embraces critical thinking skills. I should be able to use what I learnt in real-life situations, not just recalling it [LSW-16-SL-38]. |
|  | Effective time management and self-organizing | Use my time efficiently [LSW-16-SL-06]. Work in SOM is too much so one should be organised and be more time conscious [LSW-16-SL-21]. I have learnt how to read and organise myself [LSW-16-SL-45]. Organising notes, managing timetable [LSW-16-SL-31]. The need to learn on how to organise information, I am still learning how to learn. [LSW-16-SL-01]. |
|  | Integration and linking information from multiple sources | **Integration and linking information from multiple sources:** I have to learn how to integrate information and make notes [LSW-16-SL-23]. I learnt that I need to learn how I can integrate information. Not put what I got from different sources in different places [LSW-16-SL-02]. Have the ability to integrate information [LSW-16-SL-22]. Organise and integrate information in a meaningful way [LSW-16-SL-47]. The most important thing I also learnt is that information and skills integration are quite essential [LSW-16-SL-34]. The most important thing I learned today is that I should be able to link information [LSW-16-SL-27]. |
|  | Using information for application and problem solving: Learning should not be about getting | **Using information for application and problem solving:** Learning should not be about getting information but also to integrate and apply the knowledge gained in real-life situations [LSW-16-SL-10]. Integrating information and being able to retain it, explain and apply it [LSW-16-SL-18] [LSW-16-SL-13]. I have learnt that learning is not all about grasping information, but it also entails its application in solving life situations [LSW-16-SL-09]. After acquiring knowledge, I should be able to apply it to solve situations [LSW-16-SL-31]. Whatever we learnt we are supposed to integrate it to solve a problem [LSW-16-SL-24]. Be able to integrate that information to solve problems [LSW-16-SL-44]. I should improve … how to integrate the information to solve a situation [LSW-16-SL-48]. I learnt that I do not only have to read with understanding but to be able to apply and relate information [LSW-16-SL-39]. |
|  | Connecting content from different disciplines and learning events | **Connecting content from different disciplines and learning events:** I also realised that the information that we learn we have to recall it when we practice. We should form links with different aspects of medicine. For example, in learning anatomy you can relate it to pharmacology etc. [LSW-16-SL-11]. The most important thing about learning is integrating what you learn to all aspects of medicine [LSW-16-SL-40]. Knowing how to organise and build a link between information obtained from various sources and its importance in the study process of the course [LSW-16-SL-32]. |

*What is still unclear? What am I anxious about? What would I still like to learn?*

Several themes emerged concerning students' areas of anxiety, lack of clarity, and what they would like to learn. These included: learning/ study methods and strategies, self-management, adapting to and managing the PBL process, information literacy, and program-related concerns. Students reported being unclear about the depth needed when studying and uncertainty about using the right learning strategy. They also suggested there was a need to acquire subject-specific learning strategies, e.g., for learning anatomy. They raised concerns about the high workload, their capacity to maintain a healthy work-life balance, and even their ability to manage time effectively. Students acknowledged the need for information literacy skills and raised concerns about not having such skills but expressing willingness to be capacitated. They were also concerned about the lack of clarity on how they will be assessed and whether the additional effort will pay off regarding better grades. Some of the students indicated that they were also experiencing challenges adjusting to PBL and were uncertain about the PBL process.

| **Table 4 Themes emerging related to what is unclear, what they are anxious about, and what they would still like to learn about learning** | | |
| --- | --- | --- |
| **Theme** | **Codes/ categories** | **Data excerpts** |
| Students were uncertain about effective strategies to adopt for learning and were willing to be capacitated on learning how to learn effectively | - Uncertainty about learning methods/strategy - Unclear about the depth to go when studying and searching for information - Need for subject-specific learning strategies - Need for effective notetaking - Willing to be capacitated concerning learning skills - Lack of information literacy skills - It is important to have information literacy skills - Willingness to acquire information literacy skills - Synthesis of information gathered and read | **Uncertainty about learning methods/strategy:** I am not sure about my style of learning and need help on how to study effectively [LSW-16-04]. How do I know I have learnt enough for me to apply to the outside world? [LSW-16-05]. A lot of slides during lecturers … I end up not knowing what to read or do at a time [LSW-16-20].  **Unclear about the depth to go when studying and researching:** At FOM we are told to understand the depth of the information and I am still curious to know what this depth is [LSW-16-42]. How deep can I go when I am researching? [LSW-16-37]. How deep should I go when I am reading [LSW-16-39]. Willing to learn the depth to go when studying and researching - I want to be an effective reader in terms of reading with depth and understanding [LSW-16-39]. It is a skill [how deep to go when reading] I would like to learn [LSW-16-39].  **Need for subject-specific learning strategies:** Anxious about learning anatomy [LSW-16-16]. The process of gaining knowledge about a subject [LSW-16-10].  **Need for effective note-taking:** How to make notes that are still understandable [LSW-16-27]. I would like to learn how to make or take notes [LSW-16-27].  **Willing to be capacitated concerning learning and information literacy skills:** I would like to learn is how I can effectively study on my own and use the different skills [LSW-16-11]. I will highly appreciate it if I could be helped with study methods that can help me to survive in this programme [LSW-16-36]. I would still like to learn how to really study many lecturers in so little time [LSW-16-18]. Learning mostly how to organise and plan for my study [LSW-16-31]. How to prepare for the second session of PBL [LSW-16-34]. Follow the right learning strategy [LSW-16-23]. I would like to learn how to acquire relevant information from different sources on my own [LSW-16-12] [LSW-16-28], instead of Google [LSW-16-17].  **Lack of information literacy skills:** I don’t know how to find relevant information and locate resources [LSW-16-03] [LSW-16-04] [LSW-16-22] [LSW-16-23], making sense out of them [LSW-16-29], and it's quite stressing [LSW-16-34].  **It is important to have information literacy skills:** It is important to find and use relevant information [LSW-16-26], find them fast enough [LSW-16-22].  **Synthesis of information gathered and read:** How to develop skills to integrate information acquired [LSW-16-37], be able to make sense of the different sources of information [LSW-16-40], guide us to have skills to integrating information learned [LSW-16-48]. |
| Students recognize time management as essential for managing their learning and maintaining a healthy work-life balance | - Time management   - Concern about time management   - Willingness to acquire time management skills - Overwhelming workload - Concerns over work-life balance | **Concern about time management:** I am concerned about my time management skills [LSW-16-33].  **Willingness to acquire time management skills:** I need time management skills [LSW-16-14]. I want to know how I can manage my time [LSW-16-23]. I need to learn how to utilise my time when researching for PBL and studying for lecturers [LSW-16-29]. I want to know how to use and manage SDL [LSW-16-22].  **Overwhelming workload:** The lecturers are fast so sometimes it’s hard to catch up [LSW-16-24]. I wonder if I will be able to cope with the excessive workload [LSW-16-01].  **Concerns over work-life balance:** What makes me nervous is the fact that we have to give up a lot of things that we did so we can have time to adequately acquire and process information [LSW-16-33]. Being able to create a balance with my other activities (social) [LSW-16-31]. I want to know how to balance medical school and other aspects of my life [LSW-16-14]. |
| Students were uncertain about how to navigate the PBL process | - Uncertainty about the PBL process and adapting to it - Challenges with the transition to PBL | **Uncertainty about the PBL process and adapting to it:** I am still finding it difficult to adapt to this system [LSW-16-46]. The style of learning seems ambiguous [LSW-16-05]. If feel that the kind of learning strategy is difficult and I am worried if I will be able to cope [LSW-16-02]. The possibility that I might not be able to adapt to this new way of learning [LSW-16-06] and I will be left behind my colleagues [LSW-16-06].  **Challenges with the transition to PBL:** I am used to studying for an exam and not application to solve a problem [LSW-16-02]. I still don’t understand the concept of student-centered learning as to me they are practically the same. We interact with the lecturer and we end up writing an exam. The concept is still the same to me [LSW-16-25]. |
| Students are concerned about how they SoM assessment processes and how they affect their overall performance in the program | - Concerns over how they will be assessed - Concerns about if their effort will pay back - Comforted by support systems in place | **Concerns over how they will be assessed:** I still don’t know how we are going to be assessed and how the lecturer is going to evaluate our progress [LSW-16-05]. How are we going to be tested as the way we use and find information is different? [LSW-16-26].  **Concerns about if their effort will pay back:** If I spend most of my time studying and reading, will I get the best outcomes I am used to? [LSW-16-30]. Will I pass my examinations or will I have just wasted my time? [LSW-16-30]. How do I make things easier for me? I am wondering if I will be able to make it because I have noticed it’s tough [LSW-16-49].  **Comforted by support systems in place:** It is good at FOM they provide learning support; I hope this continues [LSW-16-24]. |

# Phase 3: Iterative Cycles of Delivery and Refinement

## The 2017-18 Semester-long Learning Success Program

In addition to the findings from the needs assessment (Table 1), the students' feedback from the 2016-17 offering and the facilitators' reflections were used to develop the year-long 2017/18 LSP program. The program outcomes and topics are shown in Table 5. Expanding the program to span the whole year allowed us to focus on one strategy per class session, and some strategies covered several sessions. It also allowed us to add more sessions as was revealed necessary from the Phase 1 feedback, including effective teamwork, emotional intelligence, and time management.

Upon reflection, LSP facilitators acknowledged that the 2016-17 program was more theoretical than practical. In the 2017-18 offering of the LSP, the focus was on more than telling students about strategies and describing them, but also allowing students to practice them in class. For instance, when teaching the modified minute paper, students were asked to spend 15 minutes writing non-stop what they learned during the week and then reflect on the process and areas where they felt there were gaps in their learning. After the minute paper, students worked in small groups to collate their muddy points (concepts that were not clear), taught each other what they knew (from the muddy point list), and then created a list of what they still did not understand. The whole class then collated the outstanding muddy points, and the facilitator sent them to the biomedical scientists teaching the block.

Additionally, each session ended with small-group reflection followed by whole-group reflection to enable students to think through how the strategy worked for them, if it's a strategy they can use, and where they would need to improve to use it more effectively.

| **Table 5: Summary of outcomes and topics included in the 2017-18 Learning Success Program** | | |
| --- | --- | --- |
| **Primary outcome** | **Learning Outcomes** | **Topics** |
| Implementing a strategy for effective learning in a PBL curriculum | **Understanding Yourself**  Articulate the role of a student in a learner-centered environment | - Types of transition in medical school - What is learning, eight things needed to be an effective learner* - Teacher-centered vs learner-centered teaching, exploring prior and current learning contexts - Learning preferences and implications for learning |
|  | **Learning and study strategies**  Use effective study strategies for a learner-centered environment | - Learning strategies: minute paper, concept maps/ mind maps, teach and learn, focused list, memory matrix, SQ3R, KWL chart - Presentation and poster design - Reflection as a learning strategy |
|  | **Understanding others**  Use of various inventories to gain insight into oneself | - Group dynamics - Working well with others |
|  | **Self-management skills**  Skills related to managing oneself and the learning process | - Self-care (stress management, work-life balance) - Emotional intelligence - Financial literacy - Professionalism - Time management |

As the 2017-18 LSP was being implemented, several projects within the FoM were ongoing and had input into the revision planned for the 2018-19 academic year. These were:

- *A semi-systematic review of the literature*: In 2017, the team of faculty members who conducted the needs assessment during the 2013-14 academic year conducted a semi-systematic review^7^ to examine for good understanding learning development intervention programs to address learning challenges and deficits in the first year of medical school. This was done to add input informing the program's revision for the 2018-19 academic year. The following aspects were adopted from the review and integrated into the program:

1. A proactive developmental approach to learning support
2. A year-long intervention for all students with sessions conducted once a week
3. The inclusion of three components to the intervention: learning and study skills, personal development, and professional formation skills

- *The MBBS Curriculum Review*: The MBBS curriculum review report^8^ was finalized during 2017-18, and it revealed several gaps, that is, areas that are not or are limitedly addressed and should be addressed (Table 6). The theme *"Ethics and Professionalism"* was drawn from the report to become part of LSP. Additionally, because of the highly collaborative nature of the PBL curriculum and some students' initial discomfort with group work, the theme of "*Leadership and Management"* was added to support collaborative skills and contribute to overall curriculum outcomes.

| **Table 6:**  **MBBS Review: 'Golden Threads' Curricular Gaps** | |
| --- | --- |
| - Clinical Reasoning (Q 4.6) - Community-Based Education (Q 2.8) - Complementary and Traditional Medicine (Q 2.17) - Critical Thinking (Q 2.18) - Cultural Authenticity (Q.2.12) - **Ethics and Professionalism (Q 2.20, Q 4.6)** - Evidence-Based Medicine (EBM) (Q 2.18) | - Health Equity (Q 2.12) - Interdependent Education (Q 2.9) - International Structures and Resources (Q 2.10) - Patient Safety (Q 2.7) - Research (Q 2.18, Q 5.4) - Transformative Education (Q 2.11) |

*Excerpted from University of Botswana Faculty of Medicine. Review of the Bachelor of Medicine Bachelor of Surgery (MBBS) program in the Faculty of Medicine

- *The student support and mentoring program:* Responding to multiple challenges students were reportedly facing (anecdotally observed by faculty members and reported by students representatives), such as stress, alcohol and drug abuse, and psycho-social and socio-economic challenges, the development of the Student Support and Mentoring program was pursued.^9^ The Learning Success Program was formalized as part of the Student Support and Mentoring Program^9^ towards the end of the 2017-18 academic year.

### Feedback on the 2017-18 Semester-long Learning Success Program

*Data collection:*

At the end of the 2018-19 academic year, the LSP facilitators sought feedback that would later be used to improve the 2019-20 LSP offering. At the last class session, willing students were invited to participate in providing feedback on the LSP. Instead of facilitating the feedback session, the LSP coordinators requested another faculty member from the Department of Medical Education to facilitate the feedback session to reduce coercion. Data were collected through written reflections using two open-ended questions:

- What has been the most important learning experience(s) from the *Learning Success Program*?
- What improvements, if any, have you made to enhance your learning in Semester 2?

*Data analysis:*

Thematic analysis was adopted^5^ to search for patterns in the data.^3,4^ It included familiarization with data, open coding^10^ to generate initial codes and categories, searching for, defining and naming themes, reviewing and consolidating the themes, and determining relationships between themes. The themes identified from the data were summarized as shown in Table 7 and Table 8.

### Summary of Findings

*Important Learning Experiences during the LSP*

Concerning important learning experiences during the LSP, students' comments related to group work, learning and study strategies, self-management, learner differences, and delivery of the program as outlined in Table 6. Students reported having learned the value of group work and getting to know their peers better. They also reported increased awareness of and benefits of having a toolkit of various learning and study methods. They also found it worthwhile to learn about time management and stress management and were concerned about work-life balance. Some students saw the LSP as a platform for engaging in mental health or mental health support. Additionally, they appreciated using introspection tools to gain insights into their learning preferences and understanding other students.

| **Table 7:**  **Summary of most important learning experiences from the Learning Success Program** | | |
| --- | --- | --- |
| **Theme** | **Codes/ categories** | **Data excerpts** |
| Students adopted self- and social-regulatory study strategies to enhance their learning | - General appreciation of learning strategy toolkit | Equipped me with learning strategies that help in terms of content, especially for PBL settings where presentations are made [LSP-2017-18-SL-04]. Other than my own strategies, I have learnt that there can be other ways to study and be more effective in Medicine [LSP-2017-18-SL-17]. Learning about study techniques particularly realizing that when studying I should establish first what I know, what I wish to know and at the end reflecting on what I have read to try and identify the areas in which I am learning [LSP-2017-18-SL-20]. |
|  | - Specific strategies – mind mapping, concept mapping, minute paper, muddiest points, reflection, KWL chart (what I know, need to know, what I have learned) | Reflection, minute paper and muddiest points [LSP-2017-18-SL-30]. Muddiest points – I must say the idea of identifying my weak point is a very good idea and by that I am be able to focus my attention in the right place [LSP-2017-18-SL-18]. It helped me figure out the study methods that work for me best, like the use of mind maps [LSP-2017-18-SL-01]. The most important experience was learning how to design and come up with concept maps and mind maps as they have helped me get through many exams [LSP-2017-18-SL-09]. The use of mind maps. We had enough practice, and we were able to adapt the way it works from the workshop. Placing of all our concepts from particular topic or theme of the week and writing it down on paper proved to show us what we know and what we lack, therefore aiding in our learning [LSP-2017-18-SL-28]. I have learnt how to organise my notes and about when I should study, and I have also learnt some note taking skills like mind mapping [LSP-2017-18-SL-23]. |
|  | - Presentation design | Learnt about presentations and the use of PowerPoint [LSP-2017-18-SL-15]. Learning about presentation slide construction was eye-opening really [LSP-2017-18-SL-25]. |
|  | - Group work | When we were learning about group forming from Dr. John, it was very interactive and opened my eyes to see that becoming part of a group is a process [LSP-2017-18-SL-32]. And also, we always did our learning in small groups first before discussing it with the whole class. This strengthened the bond between us classmates [LSP-2017-18-SL-16]. |
| Students recognized individual differences and how they can be leveraged for better learning | - Understanding others – working better with others and appreciating what they bring to the table. | I have also learnt to appreciate the characters of different people, and this has helped me especially with PBL, since I wasn't used to working with people [LSP-2017-18-SL-08]. Learning to understand that people are different and that in order for me to have a team to work with, I need to understand and appreciate those differences [LSP-2017-18-SL-17]. |
|  | - Understanding oneself – being comfortable being different and using strategies that work for oneself | Learning about what type of a student I am, as well as the best techniques that can suit me [LSP-2017-18-SL-07]. Understanding myself and the type of student I am [LSP-2017-18-SL-11]. Understanding myself and my surroundings as well, people I live with. This helped me learn better with me being aware of what I am capable of and which strategy work best for me [LSP-2017-18-SL-22]. |
| Students incorporate self-management stragies to ensure effective learning | - Emotional intelligence, e.g., impulse control | Emotional intelligence. It was very good way to learn to understand myself much better [LSP-2017-18-SL-32]. The most important learning experience was when I got to learn about emotional intelligence and self-understanding [LSP-2017-18-SL-06]. Taught me impulse control which I believe is a critical skill, especially in this new world where I am struggling with surviving together with getting through academics [LSP-2017-18-SL-05]. |
|  | - Time management | Also, about time management. I was not really good with being strict on time and managing my time well, but ever since the session, I have improved [LSP-2017-18-SL-31]. Stress and time management [LSP-2017-18-SL-27]. |
|  | - Stress management | And what I appreciated a lot is stress management workshop that I think came at the right moment when I was stressed [LSP-2017-18-SL-22]. Learnt also about the different skills to help us in our lives e.g., stress management skills, test taking skills [LSP-2017-18-SL-14]. |
| A few students were resistant to making necessary changes for effective learning in a PBL program | - Prefer not to have learning success | I honestly wish there was no Learning Success. I attended classes because my guilty conscience haunts me when I miss class. I want to be preparing for the exam, which is in a day from now, but I'm here waiting for pizza [LSP-2017-18-SL-12]. |

*Improvements Made to Learning Strategy, Especially in Semester 2*

Concerning improvements made to enhance learning using various learning and study strategies / techniques learned in the LSP, students' comments related to group work, learning and study strategies and self-management as outlined in Table 7. Students reported being more willing to join a study group and interaction better within groups. Students also reported engaging in self-management, including planning and time management strategies to reduce stress, help-seeking, increased learning, and self-care. Additionally, there were comments relating to both an open and a closed mindset towards the Learning Success Program.

| **Table 8 Improvements made to enhance learning in Semester 2 during 2017-18 Academic Year** | | |
| --- | --- | --- |
| **Theme** | **Codes/ categories** | **Data excerpts** |
| Students recognize the need to adopt a variety of approaches for effective learning at SoM | **Use of mind and concept mapping** for revision and integrating information | I do not spend time keeping lecture notes, instead I read, then write a summary in some kind of concept or mind map [LSP-2017-18-IMP-03]. Instead of writing long decent looking notes (as though they are going to be marked) I make mind maps, concept maps and little colourful summary that I enjoy making, save time and are aesthetically pleasing to me [LSP-2017-18-IMP-08]. I started using mind mapping, and I have understood that I am not going to get anywhere with organising my work, so I've now learnt to become organised [LSP-2017-18-IMP-09]. I started using mind mapping a lot and it helped me to see what I know and then build up on it with the things that I didn't really know [LSP-2017-18-IMP-04]. Using the concept map to link all that I learn in PBL, plenaries at SDL [LSP-2017-18-IMP-21]. |
|  | **PBL specific strategies** such as completing cases early, integrating case learning with plenaries and labs | Making sure my PBL research become much detailed and includes lectures [LSP-2017-18-IMP-07]. I have made efforts in finishing my PBL work 2/1 day before time so as to create more time for further studies [LSP-2017-18-IMP-15]. I have started integrating my PBL with my plenaries because at first, they seemed like to separate entities [LSP-2017-18-IMP-20]. Using the concept map to link all that I learn in PBL, plenaries at SDL [LSP-2017-18-IMP-21]. |
|  | **Pre-reading and post-reading,** e.g., completing reading for the week before the next week, reading before class to fill gaps during plenaries | Making notes after lesson [LSP-2017-18-IMP-07]. I have tried studying and taking notes ahead of time so that when I am in class, I would only focus on filling empty gaps in my learning [LSP-2017-18-IMP-15]. Review the whole week when it ends [LSP-2017-18-IMP-22]. |
|  | **A toolkit of strategies** – use of videos, questions, deep learning, paying attention to detail, reflection, KWL strategy, SQ3R, identifying learning objectives from block guide, trying best time to study | I also took time to reflect on what I learnt, see how much I understood and take areas that remained unclear I made sure I attended to them [LSP-2017-18-IMP-01]. Trying out different times of reading [LSP-2017-18-IMP-07]. Learning to identify things that I don't know so I can focus more on them and use of summarising as way of evaluating how well I understand concepts [LSP-2017-18-IMP-18]. The use of SQ3R is an imperative tool which I got to know long back in junior school but never used it since now in medical school where it was cemented. Its use in semester 2 has aided a lot though I started using it late [LSP-2017-18-IMP-19]. |
| Students see the benefit of working in groups and leverage the benefits to enhance their learning | Engaging in group work to learn from others | I improved my learning through collaborating with others in order to help one another on unclear concepts [LSP-2017-18-IMP-02]. More collaborative work [LSP-2017-18-IMP-22]. Study groups [LSP-2017-18-IMP-06]. I started studying with other people, more especially discussion of the PB, to identify what it is I may have left out [LSP-2017-18-IMP-12]. I am planning on joining a study group so that I may enhance my learning. Study groups really help as they push one to always study to prepare for when they meet and the difficult questions/concepts which I did not understand can be explained better by others [LSP-2017-18-IMP-16]. |
| Students incorporate self-management stragies to ensure effective learning | **Planning and time management** - being organised and managing time to avoid last minute studying and piled work | I stick to my plans; I do not let a day go by without doing what I planned to [LSP-2017-18-IMP-05]. Organised schoolwork to avoid accumulated work and one that allows me to have sufficient breaks [LSP-2017-18-IMP-06]. Proper management of time while doing the schoolwork by dividing my work schedule according [LSP-2017-18-IMP-06]. Tried to be efficient, avoided procrastination and did my work in time to avoid last minute rush [LSP-2017-18-IMP-10]. |
|  | **Help seeking** – seeking help from peers on things not understood. | I ask for help from my peers and I try by all means to make it clear whenever I am lost/left behind or don't understand something and that has helped me realise my potential and my weaknesses [LSP-2017-18-IMP-14]. |
|  | **Increased learning effort** – putting more effort to schoolwork | Extended study hours and less entertainment during school days [LSP-2017-18-IMP-11]. I also tried to spend much time around books despite marks challenges I have seen though I applied much effort [LSP-2017-18-IMP-02]. |
|  | **Self-care** – planning for rest | Making sure I rest, at least have 3 days in a week that I will sleep 5 hours [LSP-2017-18-IMP-07]. Avoid sleeping later even though I still had work to do. Stay up late reduces my energy during the day and lose concentration [LSP-2017-18-IMP-10]. |
|  | **Mindset**   - Open mindset – willing to learn new skills and engage with course though results are not always visible - Struggling with the course despite putting effort, nothing seems to be working | Try out a new form of studying which hasn't yielded expected results as yet [LSP-2017-18-IMP-13].  Struggling with the course: Honestly, I'm struggling in the course. Nothing is in place; I have sleepless nights everything but even worse on exam days. But sadly, bad results and I hate this course and other kids play but they get better grades than me [LSP-2017-18-IMP-17]. |

## The 2018-19 Year-long LSP

The findings of the semi-systematic literature review, the MBBS program review, and the student feedback from the 2017-18 LSP offering contributed to the evolution of five broad themes forming the core of the 2018-19 offering of the LSP (Table 9). The program facilitation continued to be more interactive and learner-centered, providing students with ample opportunities to practice learning skills in class.

Despite challenges with the increasing class size, we maintained the layout of the learning space to continue supporting small group work. In most cases, sessions started with students and facilitators reviewing learning from the previous session. This would typically be followed by clarifying learning outcomes and a short presentation explaining the day's strategy. Depending on the strategy, students would work individually, in pairs, in small groups, and then with the whole class. Students would practice the skills during each session and reflect on them with the small group and the entire class.

| **Table 9: Broad themes, learning outcomes and topics covered in the year-long Learning Success Program** | | |
| --- | --- | --- |
| **Topic and overview** | **Learning outcomes** | **Topics covered** |
| **Understanding yourself**  Use of various inventories to gain insight into oneself | Assess their learning style, approach to learning, and readiness for self-direction  Reflect on their learning styles and approaches and role as students in a learner-centered environment | Role as a learner in PBL, learning styles and preferences, self-directed learning readiness |
| **Learning and study skills**  Use of various learning skills and strategies | Use effective learning and study strategies for effective and productive learning in a learner-centered environment  Develop and deliver effective presentations and display professional demeanor during presentations | Reading and comprehension, e.g., SQ3R, learning anatomy, mind/concept mapping, muddiest point, minute paper, reflection, test-taking, presentation and poster design and delivery  Information literacy offered by the University of Botswana Library throughout the five years of the MBBS program |
| **Self-management**  Skills related to managing oneself and the learning process | Use productive self-management skills and strategies for managing themselves and their learning | Goal setting, time management, emotional intelligence, stress management. |
| **Leadership and management**  Skills related to understanding others, working effectively in teams, leading teams, and managing projects | Display professional collaborative and leadership behavior when working with others. | Understanding others, effective collaboration, giving and receiving feedback |
| **Professionalism and ethics**  Skills related to professional and ethical behavior and demeanor | Summarize the expected professional standards for doctors and medical students and roles played by regulatory bodies. | Four principles of bioethics, professional demeanor (Botho) |

### Feedback on the 2018-19 offering of the Learning Success Program

*Data collection:*

At the end of the 2018-19 academic year, the LSP facilitators sought feedback that would be later used to improve the 2019-20 LSP offering. At the last class session, willing students were invited to participate in providing feedback on the LSP. Instead of facilitating the feedback session, the LSP facilitators requested another faculty member from the Department of Medical Education to facilitate the feedback session to reduce coercion. Data were collected through written reflections using two open-ended questions:

- What has been the most important learning experience(s) from the *Learning Success Program*?
- What improvements, if any, have you made to enhance your learning in Semester 2?

*Data analysis:*

Thematic analysis^5^ was adopted to search for patterns in the data.^3,4^ It included familiarization with data, open coding^10^ to generate initial codes and categories, searching for, defining and naming themes, reviewing and consolidating the themes, and determining relationships been themes. The themes identified from the data were summarized as shown in Table 9 and Table 10 below.

### Summary of Findings

*Important Learning Experiences during the LSP*

Concerning the most important learning experiences during the LSP, students' comments related to group work, learning and study strategies, self-management, learner differences, and delivery of the program as outlined in Table 9. Students reported having learned the value of group work and getting to know their peers better. They also reported increased awareness of and benefits of having a toolkit of various learning and study methods. They also found it worthwhile to learn about time management and stress management and were concerned about work-life balance. Some students saw the LSP as a platform for engaging in mental health or mental health support. Additionally, they appreciated using introspection tools to gain insights into their learning preferences and understanding of other students.

| **Table 10:  Summary of most important learning experiences from the Learning Success Program** | | |
| --- | --- | --- |
| **Theme** | **Codes/ categories** | **Data excerpts** |
| Students see the benefit of working in groups and leverage the benefits to enhance their learningics and group learning skills | Effective group function – working well with others | Working well with others [2018-19-LSP-SF-15]. Working together in small groups [2018-19-LSP-SF-27]. |
|  | Connectedness - getting to know peers through groups work | Teamwork and understanding others [2018-19-LSP-SF-31]. It helped me get to know more about other people [2018-19-LSP-SF-03]. I learnt that I do not always have to stick to myself and that I should learn to collaborate and work well with others [2018-19-LSP-SF-18]. |
|  | Collaborative learning – working with others is valuable and vital | Learning through group works/teams was very helpful [2018-19-LSP-SF-03]. It also helped me better express my ideas to others [2018-19-LSP-SF-03]. Group discussions are really vital [2018-19-LSP-SF-26]. Learning how to open up and communicate well with others [2018-19-LSP-SF-33]. My impact and opinion in a group [2018-19-LSP-SF-16]. |
| Students recognize the need to adopt a variety of approaches for effective learning at SoM | Awareness of various learning strategies | The workshops help us understand effective ways of studying [2018-19-LSP-SF-02]. I learned better and effective ways to study [2018-19-LSP-SF-09]. Learning about new learning skills and how to implement them [2018-19-LSP-SF-12]. Learning new study strategies [2018-19-LSP-SF-25]. Study skills [2018-19-LSP-SF-07]. Many learning strategies [2018-19-LSP-SF-34]. |
|  | Benefits of the use of learning strategies | Improving my performance through strategies we learned and also being able to boost my confidence [2018-19-LSP-SF-05]. Studying techniques which really helped me to cope with the great amount of information [2018-19-LSP-SF-13]. |
|  | Specific learning and study strategies: planning and setting learning goals, presentation skills, reading and revision, reflection | **Planning and setting learning goals:** Learn to organize each study session, plan your day [2018-19-LSP-SF-32]. To work when you are most efficient during the day [2018-19-LSP-SF-23].  **Presentation skills:** Good presentations and speaking skills [2018-19-LSP-SF-26]. Good speaking skills [2018-19-LSP-SF-17].  **Reading and revision:** Strategic reading [2018-19-LSP-SF-26]. Fun reading skills [2018-19-LSP-SF-06]. Keeping notes in order [2018-19-LSP-SF-12]. KWL chart [2018-19-LSP-SF-37]. Minute paper [2018-19-LSP-SF-35].  **Reflection:** The self-reflection lesson at the beginning of the second semester [2018-19-LSP-SF-30]. Always introspect and recollect yourself, understand what works best for you and do exactly that [2018-19-LSP-SF-08]. I learnt I should reflect back after every semester to see what I can improve on [2018-19-LSP-SF-09]. |
| Students incorporate self-management strategies to ensure effective learning | Time management | Time management and planning [2018-19-LSP-SF-04] [2018-19-LSP-SF-06] [2018-19-LSP-SF-11]. Always creating a to-do list to help me complete my assigned work properly [2018-19-LSP-SF-14]. Lastly, I learnt about time management [2018-19-LSP-SF-12]. |
|  | Stress management | Learning stress management skills [2018-19-LSP-SF-22] [2018-19-LSP-SF-15] [2018-19-LSP-SF-26] [2018-19-LSP-SF-29] [2018-19-LSP-SF-15], ways of dealing with the stress and the pressure from the course [2018-19-LSP-SF-02]. It has also taught me the importance of not stressing over every small thing [2018-19-LSP-SF-09]. How to deal with depression [2018-19-LSP-SF-38]. |
|  | Work-life balance | How best we can manage our lifestyles combined with medical school [2018-19-LSP-SF-12]. Personal skills [2018-19-LSP-SF-12]. How to cope with the medical course and being a medical student [2018-19-LSP-SF-12]. Don't work hard but work smart [2018-19-LSP-SF-24]. Make sure you get enough time to rest [2018-19-LSP-SF-32]. |
|  | A platform for engaging around mental health/ mental health support | Having psychologists and counsellors coming to lectures and teaching us way to cope with challenges experienced in medical school [2018-19-LSP-SF-10]. Being given a chance to air out our views freely [2018-19-LSP-SF-10]. |
| Students recognize individual differences and how they can be leveraged for better learning | Insight into myself as a learner | The part where we learnt about the type of learners we are [2018-19-LSP-SF-19]. Learning and understanding myself as a student and as a person especially in the transition into medical school [2018-19-LSP-SF-16]. Learning about our personalities [2018-19-LSP-SF-20]. |
|  | Insight into others | Understanding others [2018-19-LSP-SF-16]. I liked when we learned about our different personalities and how each can help with making decisions in a team [2018-19-LSP-SF-01]. When we learned about our different personalities [2018-19-LSP-SF-19]. That people have different personalities [2018-19-LSP-SF-22]. |
| Students preffered a relaxed interactive learning environment for facilitting the LSP | Preference for an interactive approach the LSP | Interactive learning [2018-19-LSP-SF-21]. Games and practical work [2018-19-LSP-SF-36]. Being able to learn soft skills and not constantly thinking about medical school [2018-19-LSP-SF-28]. Engaging with the program [2018-19-LSP-SF-11]. |

*Improvements Made to Learning Strategy, Especially in Semester 2*

Concerning improvements made to enhance learning using techniques learned in the LSP in semester 2, students' comments related to group work, learning and study strategies, self-management, and wellness are outlined in Table 10. Students reported being more willing to join a study group and improved interactions within groups. Students also reported engaging in self-management and wellness strategies such as better stress management and better handling of failure. Students also reported better time management strategies such as planning and goal setting and increased study time.

| **Table 11: Improvements made especially in Semester 2 of 2017-18 Academic Year** | | |
| --- | --- | --- |
| **Themes** | **Categories** | **Codes** |
| Students see the benefit of working in groups and leverage the benefits to enhance their learning | Engaged in study groups – joined and involved in study groups | Joined study groups [2018-19-LSP-SF-04]. I started revising with other people a few days before the exam, which really helped me because I could explain what I understood, and they could also target and explain better on my weaknesses [2018-19-LSP-SF-03]. |
|  | Improved group interactions – better interaction with peers, more willing to engage in group work | I was also able to interact well with others as I often had group discussions with fellow colleagues [2018-19-LSP-SF-02]. [Continue being] collaborative in my learning and teaching relationship with the course [2018-19-LSP-SF-26]. |
| Students recognize the need to adopt a variety of approaches for effective learning at SoM | Reading strategies: read, review, and recite; brainstorming to identify prior knowledge before reading; setting learning objectives | I read, review and recite [2018-19-LSP-SF-09]. Before reading, I brainstorm anything that I know about the topic to see which areas I am confident in [2018-19-LSP-SF-10]. Pre-reading [2018-19-LSP-SF-14] [2018-19-LSP-SF-20]. Set objectives that guide me in my learning [2018-19-LSP-SF-15]. |
|  | Integration and revision strategies: mind maps, concept maps, muddy points, revision questions | Doing mind mapping [2018-19-LSP-SF-16] [2018-19-LSP-SF-11] and concept maps helped me summarize the work given to us [2018-19-LSP-SF-11]. I learnt to use concept maps [2018-19-LSP-SF-06]. Always identified my muddiest point and then study before the other weeks [2018-19-LSP-SF-04]. I always list my muddiest points before the tests and exams so that I cover all of them before the day of the exam [2018-19-LSP-SF-09]. Do questions on every topic [2018-19-LSP-SF-09]. |
|  | Note-taking: pre-class reading and note-taking; organizing notes; summarizing information | I also write my notes before class to familiarize myself with them [pre-reading] [2018-19-LSP-SF-10]. Note-taking during class [2018-19-LSP-SF-11]. I have organized my notes [2018-19-LSP-SF-18]. I realized that recycling newly acquired information helps it to be more engraved in my memory and helps in recalling it faster [2018-19-LSP-SF-12]. Summarizing a huge bulk of information helped me understand the core objectives of the topic at hand [2018-19-LSP-SF-12]. |
|  | Test taking strategies | The use of test-taking strategies [2018-19-LSP-SF-05]. |
| Self-management and wellness | Motivation – being enthusiastic about learning | Continue being enthusiastic [2018-19-LSP-SF-26]. |
|  | Stress management – being better able to manage stress | To engage in activities as a means of de-stressing [2018-19-LSP-SF-08]. I was able to cope well with stress [2018-19-LSP-SF-02]. |
|  | Emotional intelligence - being better able to manage failure | Learned how to deal with failure and understand that there is always a next time to do better [2018-19-LSP-SF-24]. Staying true to myself [2018-19-LSP-SF-25]. |
|  | Work-life balance – resting more | Resting more [2018-19-LSP-SF-19]. |
|  | Time management: planning and goal setting, time management techniques, improved attitude towards time management, increased study time | **Planning and goal setting:** Planning and realized it saves me time by making my work easier and more organized [2018-19-LSP-SF-06]. Goal setting [2018-19-LSP-SF-14]. Set objectives that guide me in my learning [2018-19-LSP-SF-15]. I have made a study schedule and set goals that I stick to [2018-19-LSP-SF-15]. I have a schedule for each week [2018-19-LSP-SF-21] and I read during my most productive times during the day [2018-19-LSP-SF-21].  **Time management techniques:** I have decided to incorporate a method of studying known as the "pomodoro technique" to be able to read for extended periods of time [2018-19-LSP-SF-13].  **Improved attitude towards time management:** Time management is the key principle for each day [2018-19-LSP-SF-11]. I managed my time really well, and I think that was the key thing in improving my grades. I am able to cover more content in much less time than before [2018-19-LSP-SF-23].  **Increased study time:** I added more time to my study timetable because the time I had allocated was not enough [2018-19-LSP-SF-07]. |
|  | Disengaged – closed mind-set | I have honestly not made any improvements to my learning skills during the year because my learning skills have been working for quite some time, so I might as well stick to it. [no changes] [2018-19-LSP-SF-17]. |

# Reflections and Observations

- *Attendance:* Although the Learning Success Program is not compulsory, first-year students are encouraged to attend as part of the curriculum's engagement ethos. Observations from the facilitators were that students are eager to participate in these workshop sessions. During the 2016-17 and 2017-18 academic years, the attendance rate was generally at about 75%, dropping to just over 50% for sessions held closer to an exam. Most students indicated that fear of missing something made them attend. Even those who did not perceive the sessions as valuable still attended regularly.
- *Role of the LSP:* Generally, students were clear about the Learning Success Program's primary aim: to support them in acquiring and using learning and study skills in their learning in medical school and beyond.
- *Learning without the pressure of assessment:* The Learning Success Program was designed to provide students with skills that enable effective self-direction. In light of the academic stress that students were already experiencing, the program was designed to not have much work outside class sessions. Instead, students were encouraged to apply the skills that they learned in the workshop to their learning. Although there are no graded assignments for the program, facilitators need to consider ways of documenting students' progress over time.
- *Design and facilitation of the LSP:* Students have indicated their preference for interactive sessions instead of lecture-oriented sessions. They preferred "lighter" sessions [allowed them to "chill"] rather than the workload in their other learning events in the curriculum, such as games and interactive sessions.
- *Theoretical challenges to the validity of learning styles:* When the workshop first started, students' activities included assessing their learning styles using various learning style inventories. The thinking was that students would be better positioned to capitalize on their strengths and address their gaps when they know their learning preferences. Students found the use of learning style inventories helpful in bringing awareness to personal differences and understanding their peers better. They also gave them a chance to think about their learning. However, the facilitators became aware of the questionable scientific basis for learning styles. This led to reducing the time spent on learning styles and exploring tools that could serve a similar purpose of providing students with opportunities for introspection.
- *Attention to our learning:* Both program facilitators have educational training and classroom experience, but the teaching of LSP was novel and has been a learning experience. While reflecting on the process, they realized the need to keep an open mind and to continue learning and rethinking the design and facilitation of the LSP.
- *Embedding learning skills in disciplinary areas:* Although the program's vision was from faculty members teaching Biomedical and Medical Sciences, the LSP has been mainly facilitated by educationists. The team has discussed the need for faculty development to capacitate faculty members with various learning skills and how these can be embedded in their disciplinary areas. The faculty development program will start with faculty members from the Biomedical Sciences who mainly teach in the first two years of the MBBS program.

The needs assessment findings suggest that students prefer learning "learning to learn" skills within the context of disciplinary areas. Some of the strategies used in the LSP, e.g., muddy points, concept and mind mapping, can be easily used in the classroom. As the next best strategy, the facilitation team has tried to bridge this gap by using material students were learning during the week to facilitate activities during the workshop to contextualize students' learning.

- *Grappling with the appropriate outcome measure:* It has been challenging to use grades as an outcome measure for how well the LSP helps students adjust to PBL. Students have long voiced concern regarding the misalignment of MBBS assessments with learning outcomes (in the block guides), what happens in the plenaries, and what appears in tests and exams. This misalignment has also been documented in the MBBS curriculum review.^8^ As such, it does not seem that grades alone would be a fair outcome measure. There is a need for further thinking to decide on appropriate measures, e.g., pertaining to self-direction.
- *Falling grades:* Adjustment to slipping marks is a challenge for most students, and some resort to old strategies when they think new strategies don't make a difference in their grades. Some become anxious while others take the attitude of "learning over grades."
- *Social and academic transition:* There is need to take a closer look at ways to support conceptual change to help students adjust not just academically through acquisition of learning skills but also relating to how to navigate their cultural socialization to become consistent with PBL learning.
- *Teaching and learning strategy:* For the most part, small group learning seemed to work as intended. It afforded students to get comfortable working with each other and to be able to work effectively on class assignments including teaching each other some concepts. Reflection as an integral part of the teaching and learning strategy gave facilitators an opportunity to feel the pulse of the classroom and gave students an opportunity to hear from others. Active learning was also a critical element of the teaching process, giving students ample opportunities to use skills and not just hear about them.

# Conclusions

Although student adjustment seems to take at least a semester, students reported having acquired various strategies that they could use for learning in the PBL curriculum. The continuous program reviews and improvements made to the program seemed to have enhanced its effectiveness in supporting students' learning. Students indicated that they found it helpful to use tools for self-reflection and interaction with their peers. Employing group work/group learning strategies helped improve collaborative learning. Some perceived the LSP to be effective in providing them with mental health support [referring to sessions by counselors on stress management and emotional intelligence]. Most importantly, students engaged in activities that enabled them to enhance their learning, e.g., mind maps, concept maps, and test-taking strategies. Students continue to feel challenged by work-life balance. However, those who adopt self-management strategies find that being organized reduces stress and enables them to manage their time better.

# References

1. Watling CJ, Lingard L. Grounded theory in medical education research: AMEE Guide No. 70. *Med Teach*. 2012;34:850-861. doi:10.3109/0142159X.2012.704439

2. Ritchie J, Lewis J, Elam G. Designing and selecting samples. In: Jane Ritchie, Jane Lewis, eds. *Qualitative Research in Practice: A Guide for Social Science Students and Researchers*. Thousand Oaks, CA: SAGE Publications Inc; 2009:77-108.

3. Bernard HR, Ryan GW. *Analyzing Qualitative Data: Systematic Approaches*. Thousand Oaks, CA: SAGE Publications Inc; 2010.

4. Saldana J. *The Coding Manual for Qualitative Researchers*. Thousand Oaks, CA: SAGE Publications Inc; 2016.

5. Braun V, Clarke V. Using thematic analysis in psychology. *Qual Res Psychol*. 2006. doi:10.1191/1478088706qp063oa

6. Doyle T. *Helping Students Learn in a Learner-Centered Environment: A Guide to Facilitating Learning in Higher Education*. Sterling, VA: Stylus; 2008.

7. Kebaetse MB, Kebaetse M, Mokone GG, et al. Learning support interventions for Year 1 medical students: a review of the literature. *Med Educ*. 2018;52(3):263-273. doi:10.1111/medu.13465

8. University of Botswana Faculty of Medicine. *Review of the Bachelor of Medicine Bachelor of Surgery ( MBBS ) Program in the Faculty of Medicine*. Gaborone, Botswana; 2017.

9. University of Botswana Faculty of Medicine. *Faculty of Medicine Student Support and Mentoring Program*. Gaborone, Botswana; 2018.

10. Creswell JW. *Educational Research: Planning, Conducting, and Evaluating Quantitative and Qualitative Research*. 2nd ed. Upper Saddle River, NJ: Pearson Education; 2005.
